# Flow-induced oscillations of vocal-fold replicas with tuned extensibility and material properties

**DOI:** 10.1038/s41598-023-48080-x

**Published:** 2023-12-19

**Authors:** Paul Luizard, Lucie Bailly, Hamid Yousefi-Mashouf, Raphaël Girault, Laurent Orgéas, Nathalie Henrich Bernardoni

**Affiliations:** 1 Univ. Grenoble Alpes, CNRS, Grenoble INP, GIPSA-lab, Grenoble, 38000 France; 2 Univ. Grenoble Alpes, CNRS, Grenoble INP, 3SR, Grenoble, 38000 France; 3 Present Address: CNRS, Centrale Marseille, Aix Marseille Univ, LMA UMR 7031, Marseille, France; 4 Present Address: Audio Communication Group, Technische Universität Berlin, Einsteinufer 17c, Berlin, 10587 Germany

**Keywords:** Biomaterials, Mechanical engineering

## Abstract

Human vocal folds are highly deformable non-linear oscillators. During phonation, they stretch up to 50% under the complex action of laryngeal muscles. Exploring the fluid/structure/acoustic interactions on a human-scale replica to study the role of the laryngeal muscles remains a challenge. For that purpose, we designed a novel in vitro testbed to control vocal-folds pre-phonatory deformation. The testbed was used to study the vibration and the sound production of vocal-fold replicas made of (i) silicone elastomers commonly used in voice research and (ii) a gelatin-based hydrogel we recently optimized to approximate the mechanics of vocal folds during finite strains under tension, compression and shear loadings. The geometrical and mechanical parameters measured during the experiments emphasized the effect of the vocal-fold material and pre-stretch on the vibration patterns and sounds. In particular, increasing the material stiffness increases glottal flow resistance, subglottal pressure required to sustain oscillations and vibratory fundamental frequency. In addition, although the hydrogel vocal folds only oscillate at low frequencies (close to 60 Hz), the subglottal pressure they require for that purpose is realistic (within the range 0.5–2 kPa), as well as their glottal opening and contact during a vibration cycle. The results also evidence the effect of adhesion forces on vibration and sound production.

## Introduction

Phonation refers to the production of audible air pulse trains emitted by vocal-fold vibrations in the larynx. Vocal-fold vibrations are the main acoustical source of voiced sounds, such as vowels or sonorous consonants. Contrary to the case of the heart, the vocal-fold vibrations do not result from any periodic muscular activity: laryngeal intrinsic muscles drive the vocal-fold stretching (typically between $\approx$ 10–50$\%$ strain in their longitudinal direction), adduction and abduction, but their quasi-periodic oscillations emerge from “passive” fluid/structure interactions between exhaled airflow and vocal-fold tissues^1–4^. Conceptually, vocal folds can be seen as non-linear oscillators with complex regimes of vibration, responding to gradual variation of control parameters^5–9^: geometric (e.g., vocal-fold length, glottal width and thickness), mechanical (e.g., vocal-fold tension and viscoelastic properties), and aerodynamic (e.g., transglottal pressure drop and airflow). In particular, a minimum threshold value of subglottal air pressure (Phonation Threshold Pressure) is required to initiate oscillations^1^.

Since the late 1950s^10^, to overcome the limitations of in vivo testing conditions, various artificial larynges were developed to mimic human phonation in vitro, allowing an easy control and quantitative access to the physical parameters that govern vocal-fold vibrations. In recent decades, vocal-fold replicas have evolved in complexity from rigid-walled static or forced-vibrating models to deformable, human-scale replicas able to generate flow-induced self-sustained vibrations and audible sound sources (see Kniesburges et al.^11^ for a review). The first approaches based on rigid-walled replicas mostly aimed to characterize the impact of geometrical parameters on the translaryngeal pressure drop and recovery, airflow resistance and glottal jet dynamics (e.g.,^12–15^). With the achievement of the first self-oscillating replicas (e.g.,^16–22^), interest has progressively shifted to the periodic energy transfer from glottal airflow to vocal-fold structure. In particular, it was shown that the alternating convergent-divergent shape of the glottis causes a temporal asymmetry in the average wall pressure, which is critical to sustain flow-induced oscillations^16^. Much of this experimental work has also focused on the resulting vocal-fold vibratory patterns and radiated sound emissions, in order to better understand the fluid/structure/acoustic interactions of the vibrating system, although in most cases for a given vocal-fold posturing and fixed material properties.

In comparison, the specific material properties of artificial vocal folds and their effect on the fully-coupled process have been less studied to date. Several pioneering experimental works have already sought to vary the mechanical properties of artificial oscillators, by modifying the internal pressure of fluid-filled cavities in membrane-type models^19,23^, by adjusting the chemical formulation and/or processing routes of various polymers used in 3D-molded replicas, by modulating their multi-layered arrangement^22,24,25^, incorporating fibrous reinforcement^26,27^ or local surface mechanical heterogeneity^28,29^. Such studies show that these variations can be critical on the glottal jet dynamics^24^, vocal-fold closure^27^ and surface motion^22^, collision pressure^29^, phonation threshold pressure^19,22,23,25,28^ and resulting sound spectra^19,22,26–28^.

In parallel, over the past decades, a number of theoretical and numerical studies have also addressed the impact of vocal-fold mechanical properties on phonation^30,31^, using either vibrating string/beam models of vocal folds to study their natural mode frequencies^32–35^, reduced-order phonation models with simplified fluid-structure interactions (e.g.,^36–40^) or highly resolved ones commonly based on 3D finite-element methods to simulate tissues biomechanics (e.g.,^41–44^). In particular, variations in the tensile, shear and bending stiffness of the vocal folds, as well as in the stresses they undergo during longitudinal elongation (i.e., in the anterior–posterior direction), should contribute strongly to the regulation of their natural vibration frequencies^32–35^. This is ascribed to the non-linear and anisotropic mechanical properties of the native tissues at the macroscale, driven by their fibrous arrangement at the microscale^34,35^. When interacting with airflow, a slight computed change in vocal-fold stiffness can alter their eigenmodes (i.e., structure resonances) and coupling, inducing a sudden change in phonation onset frequency^36,37,40,43^, vocal-fold vibration pattern^36,37,40,43^ including glottal opening, open quotient and closing velocity^43^, airflow rate^40,43^ and sound production efficiency^36,37^. More specifically, increasing the stiffness of the vocal folds along their longitudinal direction is predicted to generate an increase in fundamental frequency, a reduction in noise production, and, under certain conditions, an increase in both the vocal-fold contact and the excitation of higher-order harmonics^39^. Finally, the influence of material anisotropy on fluid/structure/acoustic interactions has also been simulated^38,43,44^, with longitudinal stiffness parameters expected to have greater effects on glottal flows and vocal-fold vibrations than transverse stiffness parameters^43^.

Today, the development of improved vocal-fold replicas and enriched experiments is still necessary to better understand the complexity of the multiphysical couplings involved, on the one hand, and to evaluate and dialogue with the various theories and numerical models mentioned above, on the other hand. Thus, in recent years, while improving current manufacturing procedures^45,46^, the search for optimal materials^47–49^, multi-scale structures^49^ and mechanical control^50,51^ for increasingly “bio-/phono-mimetic” vocal-fold replicas is the subject of active investigation. However:

(i) Even though vocal-fold stretching is a major aspect of phonation biomechanical control^3,32–35,39,52,53^, the number of in vitro studies involving experimental models of vocal folds able to measure and control the laryngeal longitudinal pre-strain occurring before any phonatory event is very limited. To our knowledge, although several earlier models were able to tailor fold shape and/or internal tension in pre-phonatory posture^19,22,23,54^, only two published studies have presented articulated folds that allow different degrees of anterior–posterior fold lengthening to be set^26,50^.
(ii) When characterized, the matching between the mechanics of artificial replicas and those of native vocal folds has often been studied in a single loading mode, and has most generally led to comparable elastic and/or viscous properties in the linear small-strain regime solely^22,54,55^. Although these conditions allow to identify promising “bio-/phono-mimetic” candidates, they are not sufficient to validate their behavior under multiple, finite-strain loading modes such as combined tension, compression and shear, as experienced by vocal folds during pre-phonatory posturing and vibrations^56–59^.
(iii) Due to the aforementioned limitations, the impact of material properties and their strain-induced evolution on vocal-fold vibratory patterns and aero-acoustic correlates needs to be further investigated, to complement existing databases. The further evaluation of current artificial oscillators should also be tested over a wider pre-deformation range.

Therefore, this paper presents the design and experimental characterization of an original larynx replica, allowing to control both the dynamic changes of input aerodynamic parameters and the longitudinal pre-deformation of the vocal folds with adjustable material properties upon finite strains. By combining a large multiphysical experimental database with simple analytical modeling of glottal flow already used by the voice community^19,60^, it aims to better understand the influence of the mechanical properties of isotropic vocal-fold replicas on their ability to achieve flow-induced oscillations, and on the variation of associated acoustic and aerodynamic parameters.

## Materials and methods

### Articulated larynx replica with deformable vocal folds

**Figure 1:**
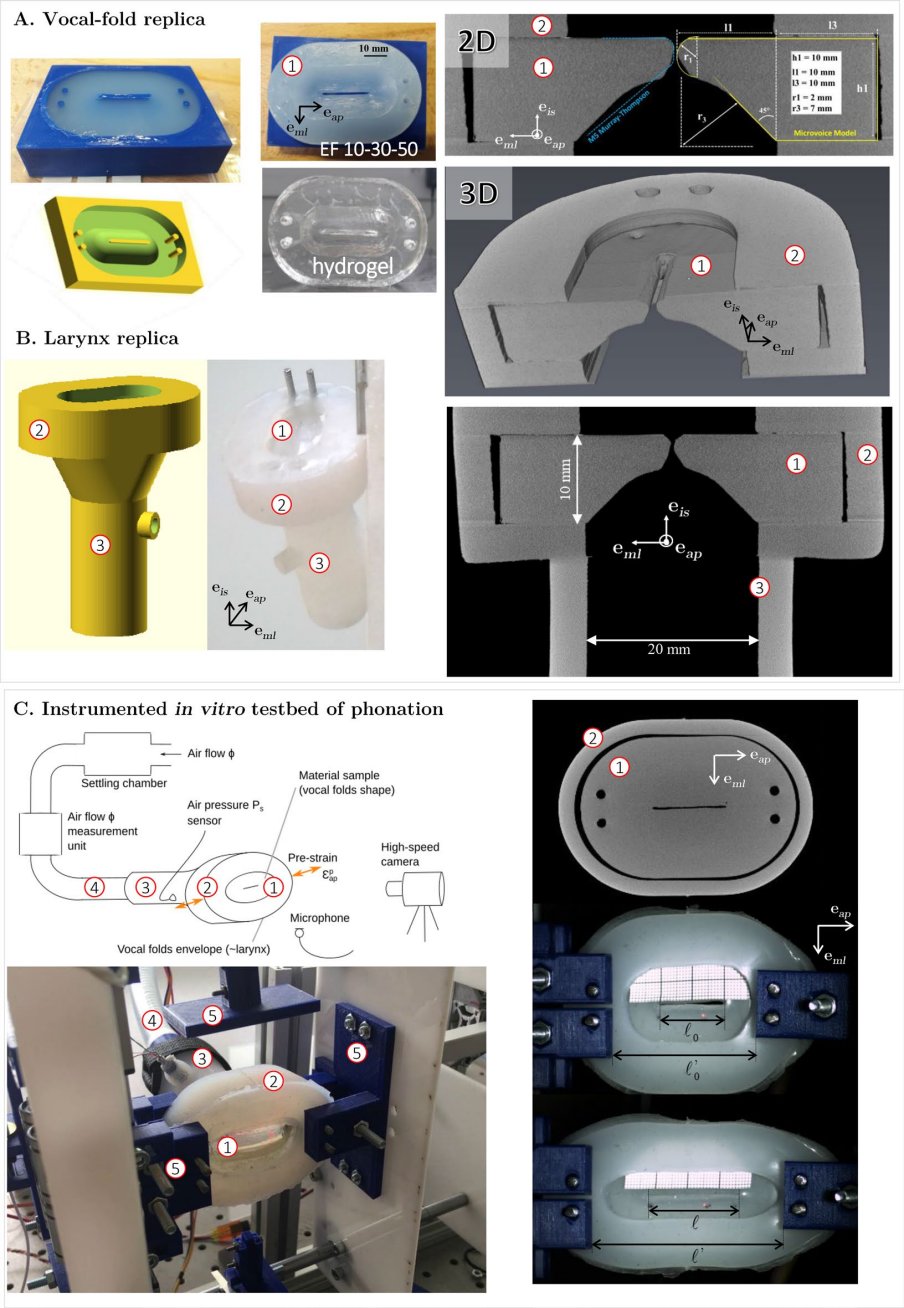
In vitro testbed of phonation. (**A**) **Vocal-fold replica:** (*from left to right*) picture of a 3D-printed mold (*in blue*), and its CAD model (*in yellow*); picture of molded replica in silicone and hydrogel; 2D vertical cross section (mid-coronal view) of a 3D vocal-fold model scanned using X-ray microtomography (voxel size $50~\upmu \hbox {m}^{3}$), geometric characterization and comparison with M5 Murray-Thomson geometry; 3D reconstructed view of half the vocal-fold replica. (**B**) **Larynx replica**: (*from left to right*) 3D CAD model and picture of the laryngeal envelope after insertion of a silicone vocal-fold replica. Mid-coronal X-ray tomographic view of the assembly. (**C**) **General view of the instrumented testbed**: (*top left*) Schematic of the setup and metrology; (*bottom left*) illustrative picture; (*top right*) X-ray microtomographic view of glottal plane (transverse view); (*middle right*) illustration of the fold at rest ($\ell _0$ = 20 mm; $\ell _0'$ = 45 mm), and in a stretched configuration (*bottom right*). ① vocal-fold replica; ② glottal stage casing, ③ subglottal tract, ④ trachea, ⑤ testbed actuators.

An in vitro testbed of human phonation was designed (Fig. 1), able to reproduce both the vocal-fold vibrations and the articulatory gestures of the larynx. It consists of a 1:1 scale vocal-fold replica inserted in a laryngeal envelope, equipped to enable the folds actuation in longitudinal stretching/compression and lateral compression. Note that this replica does not include a vocal tract. In the following, the geometry of the artificial larynx is defined in the reference anatomic frame ($\textbf{e}_{ml}$, $\textbf{e}_{ap}$, $\textbf{e}_{is}$), where $\textbf{e}_{ml}$ coincides with the medial-lateral direction, $\textbf{e}_{ap}$ with the anterior–posterior direction and $\textbf{e}_{is}$, with the inferior-superior direction.

#### Vocal-fold replica

Several single-layered and isotropic oscillators were designed and fabricated. The vocal-fold geometry (called Microvoice model) was inspired by M5 models commonly used in the literature^12,21,22^. Schematically shown in Fig. 1, the chosen geometry was a simplified version of the Murray-Thomson body model, with a circle shape for vocal-fold margin and the possibility to adjust the curvature of vocal-folds inferior part. During a prior conception phase, it was chosen as the one which enabled to sustain oscillations over the widest range of pre-strain and airflow/pressure conditions.

The folds were made up of homogeneous and soft polymers with tailored mechanical properties:

(i) A first set of folds was fabricated using a two-component (parts A and B) addition-cure silicone rubber (Smooth-On, Ecoflex™ series), as commonly used in previous in vitro testbeds of phonation^20,25,26,45,61,62^. In particular, three material candidates were selected with increasing degrees of Shore hardness (00-10, 00-30 and 00-50 respectively, based on ASTM D-2240 standards), comparable densities (1.04, 1.07, and 1.07 respectively), and processed with a 1A:1B mixing ratio by weight. They are noted EF10, EF30 and EF50 thereafter.
(ii) A second set of folds was composed of a gelatin-based hydrogel, recently optimized to approach the mechanical response of vocal-fold tissues in tension, compression and shear upon large strains^48,59^. It is based on 10$\%$ w/v porcine gelatin aqueous solution (Bloom number 300 g, Type A), cross-linked with 0.5 $\%$ mL of glutaraldehyde per gram of gelatin, to improve the strength, stiffness and ductility of the neat gel. The final relative density of the gel is close to 1.

For all cases, 20 g of uncured material was prepared in a becher for homogenization and vacuum degassing. Then, it was cast in 3D-printed molds to form a 3D structure (volume $\approx$ 16.88 cm^3^) reproducing the average morphology of healthy, adult and male vocal folds at rest (Fig. 1A), i.e., a glottis 20 mm long, 1 mm wide and 4 mm thick. Molds were previously coated with silicone grease to facilitate the demolding step. The elastomers cured at room temperature (T $\approx$ 45 °C) and relative humidity (RH $\approx 45 \%$), for 2 h for EF30 and EF50 (respectively 4 h for EF10). The hydrogel was kept at 3 °C  for 24 h before being demolded. Negligible shrinkage was observed after curing for either material.

#### Laryngeal envelope and actuators

A flexible laryngeal envelope was designed so that the processed vocal-fold replicas can be inserted and interchanged, while maintaining a seal. As shown in Fig. 1B, this three-part envelope consists of: (i) a subglottal tract attached to the air inlet tube (2 cm diameter), representing the trachea upper part (subglottal stage); (ii) an upper casing in which the vocal folds can be positioned (glottal stage); (iii) a divergent tract joining the subglottal and glottal stages. The 3D geometric assembly “vocal-fold replica + laryngeal envelope” was characterized by laboratory X-ray tomography (RX Solutions, Hamamatsu L12161-07 source), as illustrated in Fig. 1.

The assembly was connected to a series of linear servomotors (Actuonix® L16) aiming at reproducing the action of crico-thyroid tilt, i.e., vocal-fold stretching along the anterior–posterior (or longitudinal) direction $\textbf{e}_{ap}$, a key mechanism to control pitch during phonation. The junction with the motors was made thanks to three 3D-printed jaws screwed on the envelope, as illustrated in Fig. 1C.

The laryngeal envelope was molded with EF50 silicone, as a compromise between a soft and extensible material able to deform when the folds are actuated, and a material stiff enough to induce a lateral boundary condition as close as possible to fixed (ideal case mimicking thyroid cartilage stiffness). Lateral 3D-printed walls were additionally designed and integrated to the surrounding (see Fig. 1C). The vibratory capacities without and with lateral compression were pre-tested, but with limited improvements. It was therefore decided not to laterally compress the laryngeal envelope for this measurement campaign. Before vibration testing, each vocal-fold replica was manually inserted into the glottal stage, the walls of which were previously coated with silicone grease, and then attached to the laryngeal envelope and motorized jaws using Teflon®-covered screws to prevent air leakage during fold mobility.

### Mechanical characterization of the vocal-fold materials

Mechanical tests were performed on dedicated specimens made of the materials selected for the 3D vocal-fold replicas.

First, their tensile in-plane response under cyclic and finite-strain conditions was measured, following a test procedure detailed in Yousefi-Mashouf et al.^48^. Samples were cut from rectangular material plates elaborated aside from the 3D vocal-fold replicas, at an effective length-to-width ratio 5:1, with a gauge length of 50 mm and a thickness of 2 mm. Mechanical tests were carried out using an electromechanical uniaxial machine (Instron® 5944) equipped with a ± 10 N load cell (Instron®, 2530 Series). The nominal stress *P* and natural strain $\varepsilon$ were calculated from the cell force signal and displacement of the machine crosshead. Samples were subjected to 4 load-unload cycles with increasing strain amplitude up to $\varepsilon$ = 0.7, at a strain rate of 10^-2^ s^-1^. Finally, in the case of hydrogel samples, tests were conducted in a thermo-regulated atmosphere (T $\approx$ $25$ °C) and at proper hygrometric conditions ($\approx$ 98–100$\%$ RH), to protect the samples from air drying.

In a second step, to complete the mechanical database of these vocal-fold materials under multi-axial loadings, samples were also characterized in simple shear and compression, as already done on the gelatin-based hydrogel in Yousefi-Mashouf et al.^48^.

Finally, additional measurements were conducted on the same uniaxial machine, to characterize and compare the surface adhesion properties of the different materials, as already implemented on other elastic solids^63,64^. In short, two square samples of the same material were first fixed to compression plates (15 min drying time). Samples were placed in contact against each other, compressed down to a strain level of -0.20 at a rate of 10^-2^ s^-1^, and relaxed during 3 min. Then, the upper sample was pulled up to separate both bodies in contact, while measuring the force *f* resisting the separation. This force was normalized by the force registered during the relaxation step, noted $f_{relax}$.

### Aero-acoustic characterization of the vocal-fold replica

First of all, it should be noted that the vocal-fold replica and the corresponding material samples (whose mechanical behavior was tested as described above) were not only molded at the same time ($t_o$) from the same chemical preparation, but also tested on the same day, in parallel (at $t_o$ + 7 days, for a test duration varying between 2 and 5 days). This protocol has been developed and validated (data not shown) to limit as far as possible the artifacts associated with possible aging of the material^46^ or slight deviations in the processing route, and thus to be able to reliably compare the different databases acquired on the same material.

The replica was placed in the artificial laryngeal envelope, which in turn was connected to a uniform “tracheal” tube supplied with a pressurized airflow (see Fig. 1B,C) coming from a settling chamber that ensured stability of the airflow. Two major parameters were then tailored to explore fluid/structure/acoustic interactions on the testbed of phonation: (i) the vocal-fold pre-strain $\varepsilon ^p_{ap} = \ln (\ell /\ell _0)$ along the longitudinal direction $\textbf{e}_{ap}$, where $\ell$ (resp. $\ell _0$) refers to glottal length in the deformed (resp. undeformed) configuration (see Fig. 1C); (ii) the mean airflow rate $\phi$ through the larynx.

#### Instrumented testbed of phonation

The overall set-up is equipped with a number of sensors to acquire real-time data for various levels of $\varepsilon ^p_{ap}$ and $\phi$ (see Fig. 1C): an airflow measurement unit (TSI 4043), measuring $\phi (t)$ at time *t*; a pressure sensor (Kulite Xcq-093) measuring the aerodynamic subglottal pressure $P_s(t)$ relatively to ambient atmospheric pressure; a microphone (DPA 4060) calibrated by means of a sound level meter (Brüel & Kjaer 2250); and a high-speed camera (Mikrotron® MotionBLITZ EoSens®  Cube7) to visualize the vibrations of the vocal-fold replica. Using a dedicated LabView® interface (National Instruments) and the motorized control system of the laryngeal envelope, the vocal-fold pre-strain $\varepsilon ^p_{ap}$ was driven by the displacement of a series of jaws (see Fig. 1C,⑤). The airflow rate $\phi$ was driven by the degree of opening of an electromechanical valve actuator upstream in the tracheal tube replica.

Electrical signals were processed using a preamplifier/conditioning board (PXIe-1073 chassis equiped with NI PXIe-6341 and NI PXIe-4330 modules). The acquired data were processed using the LabView13 software (National Instruments). The sampling frequency was 22.05 kHz for all pressure, flow, and acoustic acquisitions. The high-speed camera images, captured at a frame rate of 1473 frames/s, i.e., three frames every 2 ms, were synchronized by the MotionBlitz software directly called in LabView, so that 100 frames were recorded at each step of increased airflow rate. The image size was 736 $\times$ 1296 pixel^2^ with a resolution of 123 pixels/cm. Repeated measurements of a known distance yielded a coefficient of variation (ratio of standard deviation over mean value) of 0.1 mm for a length, i.e. $0.1\,\hbox {mm}^{2}$ for a surface.

#### Testing protocol

Prior to the experiments, ranges of airflow input values allowing for vocal-fold self-oscillation to occur were determined, according to the pre-strain conditions. Then, a series of experimental rounds were recorded. An experimental round consisted in setting a given value of fold pre-strain $\varepsilon ^p_{ap}$. The airflow rate $\phi (t)$ was then increased by steps, from its minimum value for oscillation (typically $\phi _{min} \approx 0.3$ L/s) to a maximum value related to $100\%$ valve opening ($\phi _{max} \approx 3.5$  L/s). Each quasi-steady step lasted 4 s, during which the sensor values were recorded. For each tested vocal-fold replica material, the experimental procedure was repeated from rest configuration ($\varepsilon ^p_{ap}$ = 0) up to the maximal deformation authorized by the setup ($\varepsilon ^p_{ap}$ within 0.25–0.30 depending on the chosen material).

#### Data processing

**Figure 2:**
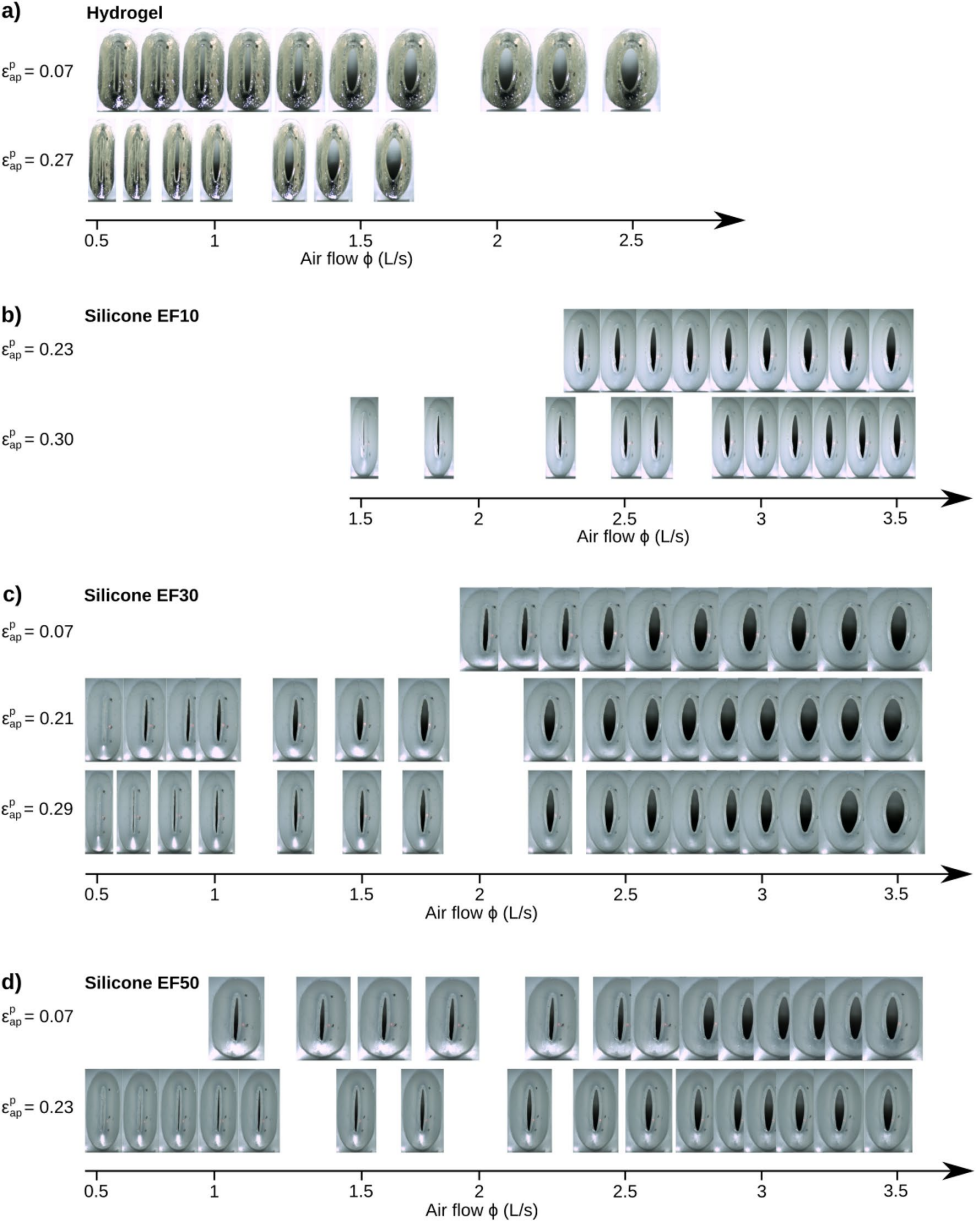
Pictures of the maximal glottal opening $A_g^{max}$ achieved during oscillation for various mean airflow rate and pre-strain values with (**a**) Hydrogel, (**b**) EF10, (**c**) EF30, and (**d**) EF50 silicone models. This illustrates specific series showed in Fig. 5. The blank spaces between pictures correspond to airflow rate values at which no measurement was performed. Corresponding videos for all pre-strains and increasing airflow sequences are provided as supplementary material. A twin figure with subglottal pressure in *x*-axis is provided as supplementary material (Fig. S1).

**Figure 3:**
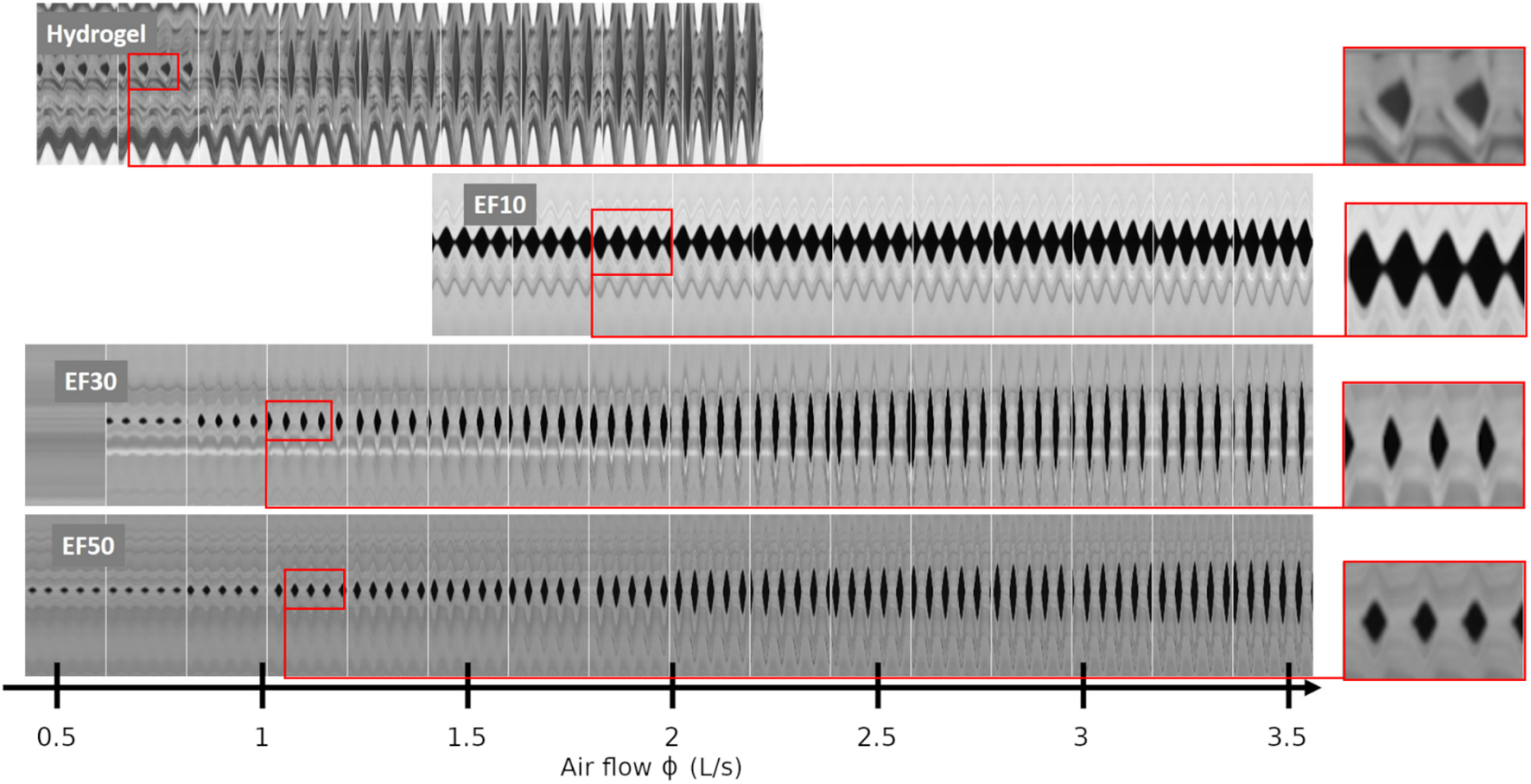
Kymographic visualization of the vibration of vocal-fold replicas composed of the four different materials, with respect to increasing airflow steps. Pre-strain conditions are: $\varepsilon _{ap}^p =$ 0.24 for Hydrogel, 0.27 for EF10, 0.26 for EF30, and 0.20 for EF50. Corresponding videos for each sequence are provided as supplementary material.

**Figure 4:**
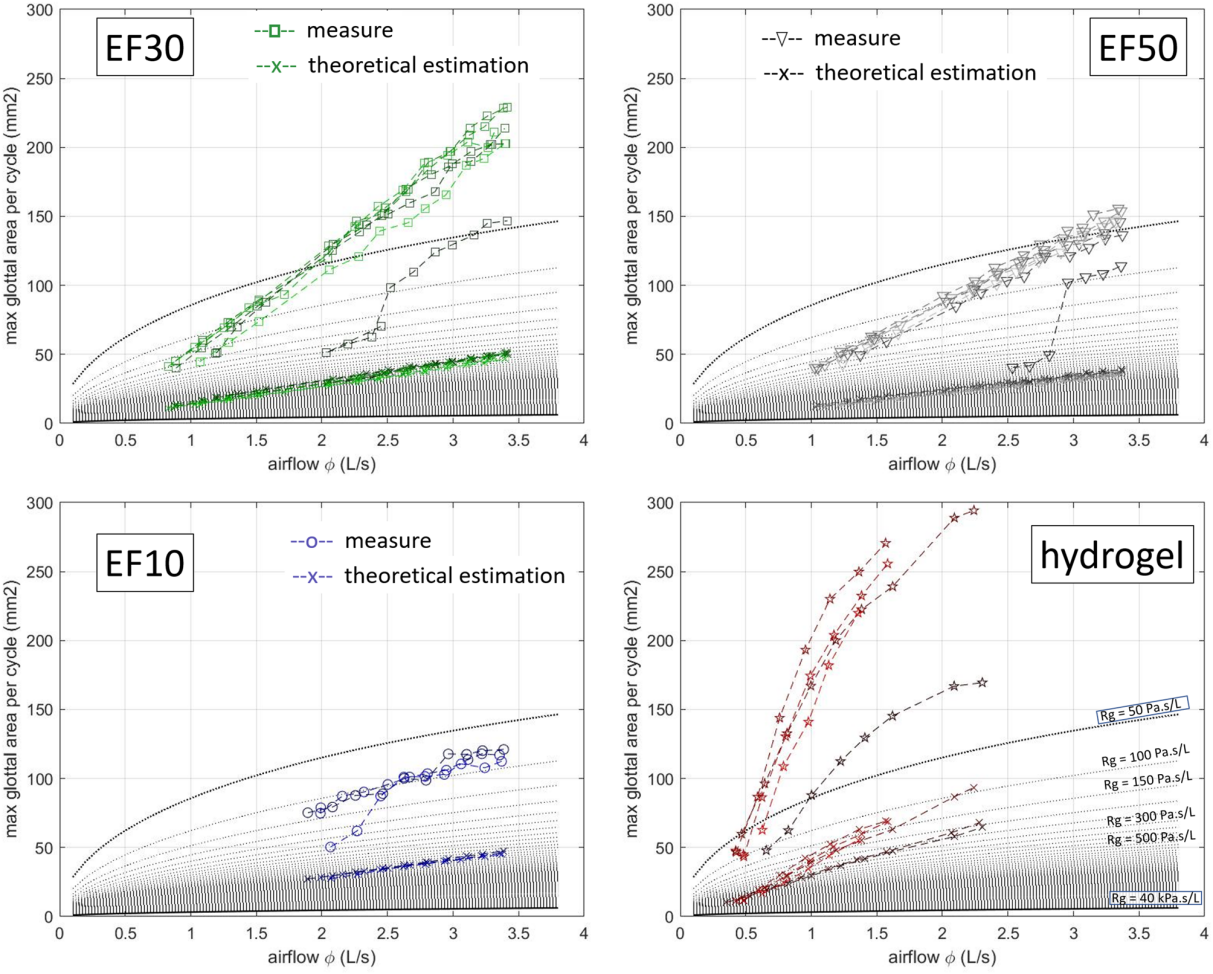
Maximum glottal area per cycle $A_g^{max}$ as a function of airflow $\phi$: comparison between experimental measurements and theoretical predictions ($\times$ markers) of steady Bernoulli’s equation (Eq. 2), knowing experimental subglottal pressure $P_s$ and airflow rate $\phi$. The colors correspond to the different materials, chosen in line with Figs. 5, 6 and 7. The dotted gray lines represent theoretical abacuses (Eq. 2) of constant glottal flow resistance $R_g$ ranging from 50 Pa s/L (dotted dark line) to 40 kPa s/L (plain dark line).

**Figure 5:**
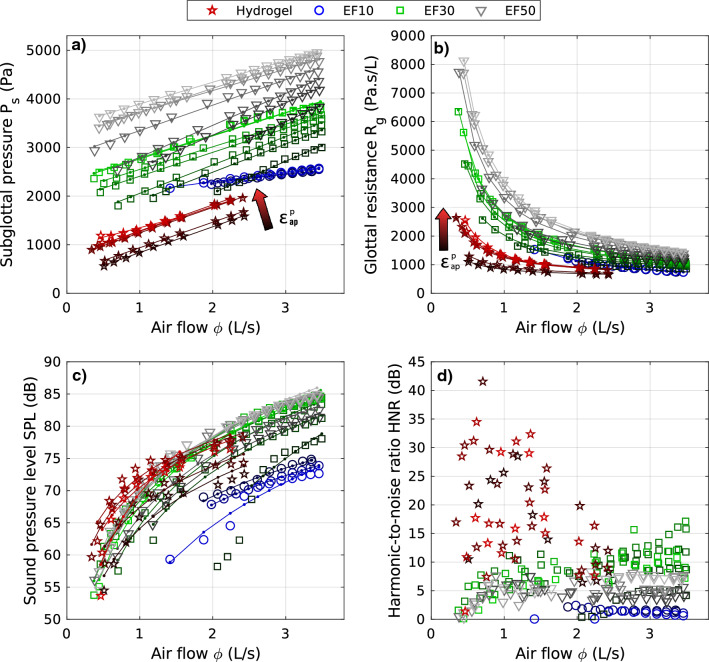
Aerodynamic (**a**- subglottal pressure, **b**- glottal flow resistance) and acoustic (**c**- sound pressure level, **d**- harmonic-to-noise ratio) parameters as a function of airflow, for each material and all pre-strain levels $\epsilon _{ap}^p$ along the anterior–posterior direction. Dark to light colors stand for increasing $\varepsilon _{ap}^p$ values presented in Table S1. Markers stand for experimental data points while solid lines represent the best empirical fits adjusted to the data for a given $\varepsilon _{ap}^p$ (see Table S1 for the fitting coefficients). Values of fundamental frequency of oscillation as a function of airflow are provided in Fig. 6a.

**Figure 6:**
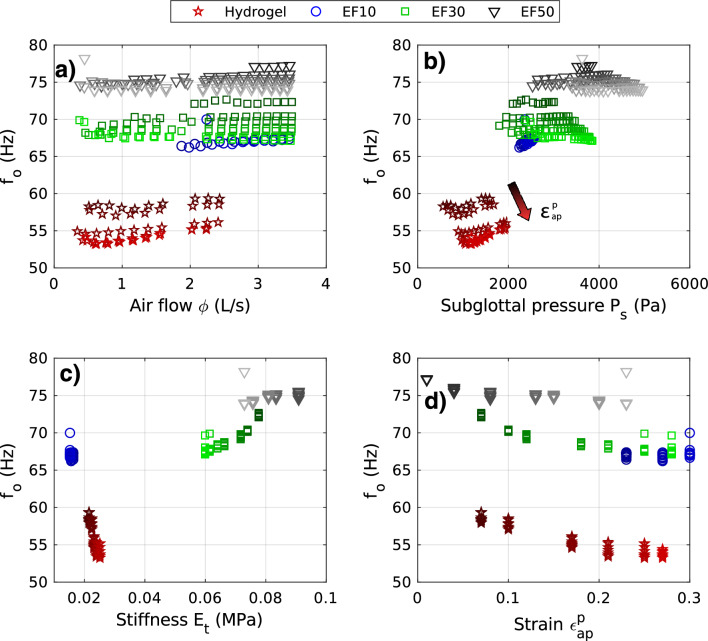
Fundamental frequency of oscillation against various parameters that influence the phonation characteristics. Dark to light colors indicate an increase of pre-strain $\varepsilon _{ap}^p$.

Each round was decomposed into a series of successive quasi-steady states corresponding to increments of airflow increase, for which several time-varying aero-acoustic parameters were averaged over the 4-s time window, yielding the following mean values: airflow rate $\phi$ and subglottal air pressure $P_{s}$, oscillation frequency $f_o$ calculated from the audio signal using the YIN-auto-correlation method^65^, the calibrated sound pressure level *SPL* in dB without specific weighting, and the harmonic-to-noise ratio *HNR* that quantifies the dynamics of the temporal harmonic signal. The glottal flow resistance $R_g$ was estimated as the ratio of the overall mean pressure drop $\Delta P$ through the larynx to the associated mean airflow rate, such as $R_g = \Delta P / \phi$^10,66–69^. The units of these quantities are specified in Table S1 and in Figs. 2, 3, 4, 5 and 6.

High-speed images were edited and analyzed using the Glottis Analysis Tools 2020 software^70^, which enables glottal area contour detection with a threshold-based region growing approach, combined with trained neural networks. When required, automatic segmentation was manually corrected to account for minor detection errors on one glottal cycle per each quasi-stationary step. Contrary to other recent visual techniques that aim at determining vibration modes of vocal folds^71^, this image analysis software provides the time-varying glottal area, $A_g(t)$. Maximum values achieved over a cycle were then extracted at each quasi-steady step, and noted $A_g^{max}$. The pre-strain $\varepsilon ^p_{ap}$ actually applied to each vocal-fold replica was measured on these images (one value per experimental round). In order to obtain a dynamic view of the median oscillatory motion of the replica, kymograms^72^ were also plotted in Matlab®. The kymographic line was selected on a first image in the middle part of the glottis and perpendicular to the anterior–posterior glottal axis. It was then plotted for the whole high-speed sequence as a function of time. While kymograms do not allow to visualize glottal vibrations along the whole glottal length, they provide a detailed representation of vocal fold dynamics at the selected position on the glottis.

### Analytical modeling of laryngeal flow

A simple analytical model based on Bernoulli’s principle for fluid flow was used to compare experimental measurements with theoretical relationships between subglottal air pressure $P_{s}$, airflow $\phi$ and glottal area $A_{g}$. This modeling approach was previously applied to understand the aerodynamic pressure distributions along the glottal channel^19,44,60,73–77^. Assuming perfect incompressible fluid and quasi-steady flow conditions ($d\phi /dt \approx$ 0), Bernoulli’s equation applied between sub- and supraglottal stages yields to:

1 $$\begin{aligned} P_{s}+ \frac{1}{2} \rho \Big (\frac{\phi }{A_{s}}\Big ) ^{2} = \frac{1}{2} \rho \Big (\frac{\phi }{A_{sep}}\Big ) ^{2}, \end{aligned}$$

where $\rho \approx 1.20$ kg/m^3^ is the density of air at $22$ °C, $A_{s} = 3.14$ cm^2^ is the cross-sectional area of the channel flow in the trachea where subglottal pressure $P_{s}$ is measured, and where $A_{sep}$ is that at the point of separation of the glottal jet from the walls, where relative air pressure can be neglected^60,76^. Under these assumptions, the mean pressure drop $\Delta P$ governing the aerodynamics from the glottal inlet into the trachea to the point of flow separation corresponds to the mean subglottal pressure $P_s$. Previous research established the relationship $A_{sep} {\approx } 1.2~A_g$, based on Liljencrants’ ad hoc criterion, i.e., a semi-empirical model used as an alternative to a boundary-layer separation theory^19,77^. Therefrom, theoretical estimations of glottal area $A_g$ and glottal flow resistance $R_g$ can be derived from Equation (1) as follows:

2 $$\begin{aligned} A_g~{\approx }~ \frac{1}{1.2}~A_{sep} = \frac{1}{1.2} \Big ( \frac{2}{\rho } \frac{P_s}{\phi ^2} +\frac{1}{A^2_s} \Big )^{-1/2} \qquad \text {and} \qquad R_g = \frac{P_s}{\phi } ~{\approx }~ \frac{1}{2} \rho \phi \Big (\frac{1}{(1.2~A_{g})^{2}} -\frac{1}{A_{s}^{2}}\Big ) \end{aligned}$$

## Results and discussion

### General trends

Regardless of the specific material features of each vocal-fold replica, common qualitative trends were observed in their overall geometrical and aero-acoustical behavior during oscillation.

#### Geometrical variations

Figures 2 and 3 illustrate the qualitative changes in glottal geometry observed on the four vocal-fold replicas during the progressive increase in airflow rate. As expected^78^, whatever the replica and its level of pre-strain $\varepsilon _{ap}^p$, greater airflow $\phi$ increases both the maximum glottal area reached during the oscillation cycle, $A_g^{max}$ (Fig. 2), as well as the amplitude of vocal-fold vibrations in the mid-glottis (Fig. 3). Such changes are also illustrated in the Supplementary Videos of both figures, e.g., on EF30-eps21.mp4 corresponding to silicone EF30 at medium pre-strain $\varepsilon _{ap}^p$ = 0.21. Besides, for all cases, Fig. 4 compares the measured values of $A_g^{max}$ to their theoretical predictions as derived from Eq. (2). As the experimental data relate to the maximum values of $A_g(t)$ over a cycle, it should be noted that the corresponding minimum value recorded for the subglottal pressure $P_s(t)$ was used here as input parameter in the model. To complete the graphs and account for the impact of this choice on the predictions, a zone of theoretical variation is also represented by abacuses, i.e. for constant values of glottal resistance $R_g$ ranging from 50 Pa s/L to 40 kPa s/L. Observed trends in measured data are reproduced at least qualitatively, with an increase of $A_g^{max}$ with airflow rate $\phi$. However, the predicted values underestimate the measured ones by a factor 2 to 10 depending on the material. Interestingly, the predicted values are in a range similar to previously reported data from measurements in humans^79^, i.e. 5 to 25 mm^2^ (see Table 1).

#### Aerodynamic behavior

Figure 5a,b shows the evolution of mean subglottal pressure $P_s$ and glottal flow resistance $R_g$ as a function of airflow rate $\phi$. Each point represents a quasi-steady state for which the replica exhibited stable self-oscillations. A similar figure plotting the glottal flow resistance $R_g$ and the airflow rate $\phi$ as a function of mean subglottal pressure is provided in Supplementary Fig. S2d–f. In addition, the ranges of the parameters measured in the current study as well as values of in vivo and ex vivo measurements from previous research are presented in Table 1.

Several trends can be highlighted:

- The present in vitro candidates are characterized by mean subglottal pressure $P_s$ within 500 Pa to 5000 Pa, as shown in Fig. 5a. These pressures are similar to those observed in ex vivo studies^80–84^ and in vitro ones^50^. For in vivo data, the range reported is 200 to 1000 Pa , with thresholds exceeding 2000 Pa at louder intensities^11,45^. The designed replicas are able to cover about 80$\%$ of the target data in the swept parameter ranges.
- A quasi-linear flow-pressure relationship is observed for all cases (Fig. 5a). The values of slope and *y*-intercept are detailed in Table S1. The rate of variation of subglottal pressure against airflow, i.e., the mean slope values, correspond to the differentiated glottal flow resistance $R_d$^81,82^ defined in the case of linear pressure–flow relationship as $P_s=R_d ~ \phi + P_0$. It is found to be rather similar across all materials ($R_d^{EF30} = 530 \pm 74$ Pa s/L, $R_d^{EF50} = 560 \pm 108$ Pa s/L, $R_d^{Hydrogel} = 502 \pm 56$ Pa s/L) except for EF10 ($R_d^{EF10} = 185 \pm 30$ Pa s/L). These quasi-linear trends between flow and pressure are consistent with several previous ex vivo measurements performed on various animal models for mid-levels of vocal-fold adduction^80–82^, yet with much lower $R_d$ values than for animal models ($R_d$ in the range $2-11$ kPa s/L)^81,82^. It is in line with observations on elastomeric vocal folds^50^.
- For all materials and pre-strain levels, the higher the airflow rate, the lower the glottal flow resistance, as described by the phenomenological model adjusted to the data (see Fig. 5b): ${R_g} ~=~ P_0 ~\phi ^{-1} + R_d$. The decrease of glottal flow resistance with increasing airflow rate can be related to the geometrical variations mentioned above, i.e. an increase in maximum glottal area and glottal width during the vibratory cycles as shown in Fig. 2. Increasing the anterior–posterior pre-strain $\varepsilon _{ap}^p$ does not change the global trend, yet slightly modifies the general level of the relationship between $\phi$ and $R_g$. At constant airflow, higher pre-strain implies higher glottal flow resistance for all materials, related to higher values of fit parameter $P_0$.

#### Acoustic behavior

Figures 5c,d and 6a show the impact of the airflow rate $\phi$ as measured on each acoustic parameter of the four vocal-fold replicas. As with aerodynamic parameters (Figs. 5a,b), each point corresponds to a time-averaged measurement over a $\phi$-step. The corresponding graphs displayed as a function of the mean subglottal pressure $P_s$ are available in Supplementary Fig. S2a–c. Illustrative audio cases can also be found in Supplementary Videos of Figs. 2 and 3 (e.g., EF30-eps21.mp4). General trends are evidenced on the main parameters commonly used to assess voice production^85,86^:

- The vocal intensity level, reflected here by the sound pressure level (*SPL*), is displayed in Figs. 5c and S2a. As expected in vivo^87,88^, ex vivo^84,89^ and in vitro^90^, an increase of *SPL* is found when increasing airflow rate and subglottal pressure. More specifically, *SPL* and $\phi$ (resp. $P_s$) are linked by a logarithmic and affine relationship such as $SPL = k_1~\log (\phi ) + \ell _1$ (resp. $SPL = k_2~\log (P_s) + \ell _2$) with $k_1$, $k_2$ and $\ell _1$, $\ell _2$ being constants that are quantified in Table S1 for each case. Interestingly, all experimental data follow almost the same curve within the measurement deviations (with a few exceptions for EF10 as well as EF30 at the lowest $\varepsilon _{ap}^p$). These typical non-linear *SPL*-$\phi$ and *SPL*-$P_s$ master curves are in line with observations already reported from in vivo measurements^87,88,91^. Furthermore, for the parameters swept in this work, as displayed in Table 1, the different in vitro materials are able to cover a reduced range of the standard target in vivo data: during human phonation, *SPL* varies between 30 and 130 dB (at a distance of 30 cm from the mouth)^2^, with a standard range within 55–80 dB and a mean of 65 dB at casual speech (untrained healthy voices)^92^.
- The tonal pitch, related here to fundamental frequency of vocal-fold oscillation $f_o$, is displayed in Fig. 6. With fluctuations of only a few Hz, the frequency $f_o$ is measured almost constant over the swept $\phi$-range as the flow rate increases (Fig. 6a). Although this $f_o - \phi$ relationship has not been found in ex vivo studies^84^, a plateau effect was observed for several models of silicone vocal folds, for which $f_o$ increased linearly with $\phi$ until it reaches a saturation point at a flow greater than $\approx$ 0.6 L/s^50^. Similar observations apply to the $P_s - \phi$ relationship as illustrated in Fig. 6b where the patterns regarding the materials as well as the pre-strain levels can be related to Fig. 6a by a scale factor on the *x*-axis. Moreover, whatever the case, the vibration frequencies measured on the four vocal-fold replicas are in the lowest range of the human voice, with values ranging between 50 to 80 Hz, i.e., within a window of $\approx$ 30 Hz (see Table 1). As a reminder, in human phonation, $f_o$ can range from about 50 Hz to more than 1500 Hz^2^, with seldom frequencies below 100 Hz in “normal” voice (modal phonation using laryngeal mechanism M1)^93,94^, and mean values of 125 Hz and 210 Hz for men and women speech respectively^2,94,95^. See Table 1 for a direct comparison of the ranges measured in the replica and humans. One can note that this low frequency range is found in laryngeal mechanism M0 or vocal fry^93,94^ for which the folds are very thick and passive, with limited contraction of thyro-arytenoid muscles. To better understand such global low-pitch measurements on the replicas, we first investigated possible acoustic coupling with the subglottal tract, likely to drive the vibration frequency of artificial vocal folds in some cases^17,96,97^. The first resonance frequency of the subglottal tract in our experiment was estimated around 14 Hz (the open-closed tube between the settling chamber and the vocal folds being 6.2 m long). Hence, harmonics of subglottal tract resonances might appear in the $f_o$ range of the present study, and possible interactions are not totally excluded^97^. However, their influence would certainly be minimal given the high length of the subglottal tract. Instead, our low $f_o$-recordings can be explained by the geometric choices made for this first series of articulated replicas, i.e., a glottal geometry at rest ($\varepsilon _{ap}^p$ = 0) in the upper anatomical limit ($\ell _0$ = 20 mm), and fixed boundary conditions rather far from the glottal extremities ($\ell _0'$ = 45 mm; Fig. 1).
- Finally, the resulting sound quality also varies with $\phi$, as reflected by the harmonic-to-noise ratio (*HNR*) reported in Fig. 5d. Higher values of *HNR* reflects a clearer, less noisy sound quality. Thus, a general trend emerges for the silicone vocal folds: the higher the airflow, the higher the sound clearness. It is noted that the hydrogel does not behave like silicones, showing no specific trend against $\phi$ and producing globally higher *HNR* values. Table 1 shows that the silicone replicas match the lower range of human production while the hydrogel replica reaches the upper range and even higher.

**Table 1:** Aerodynamic, acoustic, geometric, and mechanical parameters measured in the present vocal-fold replicas for different materials (min–max values).

	EF10	EF30	EF50	Hydrogel	Human vocal folds
Flow rate $\phi$ (L/s)	1.4–3.4	0.37–3.5	0.38–3.4	0.34–2.4	0.1–0.3^98,99^ (IV)
Subglottal pressure $P_s$ (Pa)	2160–2570	1800–3850	2530–4950	560–1960	200–2000^95^ (IV)
Glottal resistance $R_g$ (kPa s L^-1^)	0.7–1.5	1.0–6.3	1.3–8.1	0.7–2.6	4–14^99^ (IV)
Fundamental frequency $f_o$ (Hz)	66–70	67–73	74–77	53–60	50–1500^2^ (IV)
Sound pressure level *SPL* (dB)	59–74	53–84	55–85	54–78	30–130^2,92^ (IV)
Harmonic-to-noise ratio *HNR* (dB)	0.5–2.4	0.4–17.1	0.5–8.3	1.4–41.6	3–25^100–103^ (IV)
Maximum glottal area $A_g^{max}$ (mm^2^)	78–121	27–228	23–155	31–322	5–100^57^ (EV)
Pre-strain $\varepsilon_{ap}^p$	0.23–0.30	0.07–0.29	0.04–0.23	0.07–0.27	0–0.40^57,58,104,105^ (EV)
Tensile tangent modulus $E_t$ (MPa)	0.015–0.024	0.057–0.086	0.068–0.107	0.022–0.033	0–3.30^56,59,105–107^ (EV)

**Figure 7:**
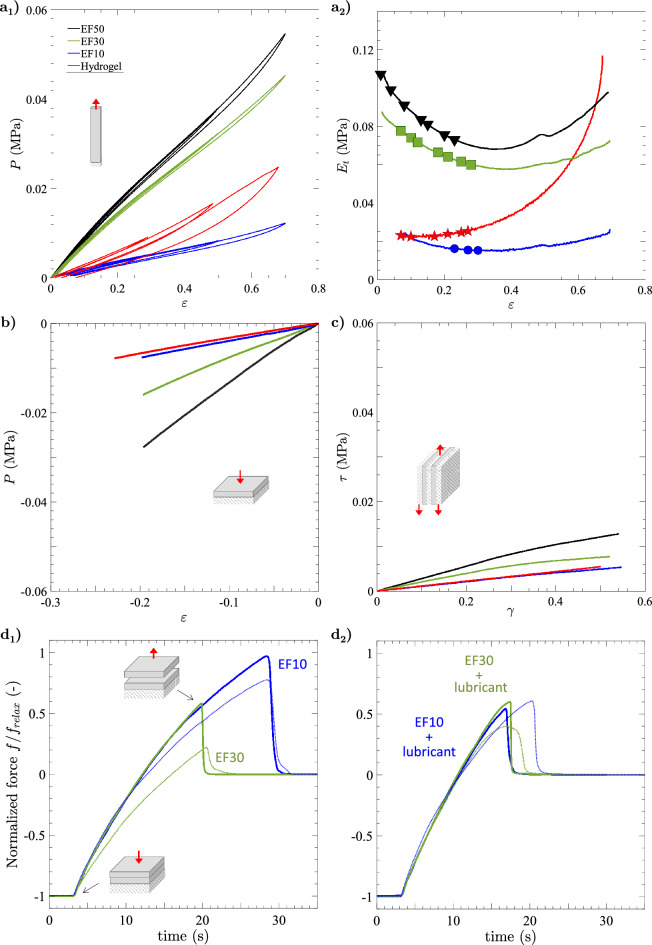
(**a**) Tensile behavior of the four materials used for the vocal-fold replica during quasi-static and cyclic loading conditions: (**a**_1_) stress–strain response (*P*, $\varepsilon$), (**a**_2_) corresponding tangent modulus $E_t$. For each material, the symbols highlight the pre-strain $\varepsilon _{ap}^p$ which was applied to the vocal-fold replica and for which self-sustained oscillations could be observed (see detailed values in Table S1). (**b**) Same as (**a**), in simple compression. (**c**) Same as (**a**), in simple shear: stress–strain response ($\tau$, $\gamma$), as defined in Yousefi-Mashouf et al.^48^. (**d**) Results of adhesion testing on EF10 and EF30 samples: (**d**_1_) after curing without any additional coating; (**d**_2_) coated with lubricant after curing (silicone oil). Dotted line illustrates the typical repeatability of each test.

Comparison with physiological values reported in the literature from in-vivo (IV) or ex-vivo (EV) measurements in humans. The extreme $E_t$ values given for synthetic materials correspond to measurements evaluated over a range of tensile strains up to 0.4 (see Fig. 7a_2_). The values reported for human vocal folds correspond to strain levels within this range, albeit lower or equal, and acquired on the whole multi-layered tissue or on individual sub-layers.

### Impact of the vibrating material

#### Mechanical properties of the different vocal-fold materials

The mechanical stress–strain behavior of the four materials is reported in Fig. 7, under finite-strain tension (a_1_), compression (b) and shear (c). Figure 7a_2_ details the evolution of the tensile tangent moduli $E_t = dP/d \varepsilon$ with the applied strain $\varepsilon$ (for the last unloading path). Regardless of the loading mode, two groups of materials stand out, and can be classified from the softest to the stiffest (from rest to $\approx$ 0.6 strain): EF10 silicone and gelatin-based hydrogel on the one hand, EF30 and EF50 silicones on the other hand. In addition, note that the overall mechanical behavior of EF10 silicone and hydrogel are particularly close up to $\varepsilon$ $\approx$ 0.3, with quasi-superimposed responses in compression and shear, as well as similar low-stress levels (up to $\approx$ 0.006 MPa) and near-constant $E_t$ values ($\approx$ 0.02 MPa) in tension.

Furthermore, EF10 silicone stands out from the other materials for its interface properties, being the only system to feature a “tacky” surface. Indeed, Fig. 7d_1_ shows that the dimensionless force measured to separate two EF10 silicone samples is higher than that obtained to separate two EF30 silicone samples. Adhesion measurements demonstrate that the separation of samples in EF10 occurs well after the separation of samples in EF30 ($\approx$ 10 s delay). Note that the stickiness of E10 silicone can be eliminated by lubricating its surfaces with silicone oil, as demonstrated in Fig. 7d_2_.

#### Impact on the onset of the vocal-fold vibrations

The influence of the material properties of the vocal-fold replicas on their flow-induced vibrations is first highlighted in Fig. 7a_2_. The symbols illustrate the cases where a stabilized oscillation was observed: for each material, below the pre-strain values $\varepsilon _{ap}^p$ marked by these symbols, no vibration was observed. The highest value, on the other hand, indicates the maximum tested during the campaign (oscillations might occur beyond these values). More specifically, none of the replicas could oscillate in their undeformed configuration: they had to be slightly pre-stretched up to $\varepsilon _{ap}^{p} \approx$ 0.05 ± 0.02 for fluid/structure interactions to give rise to sustained vibrations, except for E10 replica which only began to oscillate at a much higher level of strain, i.e., for $\varepsilon _{ap}^{p} \approx$ 0.23. Such a discrepancy is due to the specific sticky interface of the EF10 silicone, which has a major impact on the upstream air flow required to lift the folds from each other and generate sustained vibrations (see below). In addition, looking at both stiffer smooth-surface replicas (EF30, EF50), it is also interesting to note that the stiffer the material, the lower the pre-strain required to induce vibrations.

#### Impact on the sustained vibratory pattern

Once established, the database shows a strong impact of the chosen material on the type of vibrations allowed by the vocal-fold replicas. From a qualitative point of view, for given values of airflow and pre-strain levels, Figs. 2 and 3 show geometric differences in the glottal opening and vibratory amplitudes achieved during the oscillation cycle, which are much higher for the hydrogel replica than for silicone candidates. This is also particularly evidenced on Supplementary Videos provided with such figures (e.g., see 4mat−airflow2Ls.mp4 comparing the four oscillators at comparable airflow $\phi \approx$ 2 L/s). Clear discrepancies are also noticeable in the contact properties between the vocal folds, with respect to collision duration as well as fold opening and closing speed, i.e., key parameters in voice quality^94,108^. More specifically, the kymographic visualization of the high-speed sequences in Fig. 3 and Supplementary Videos highlight:

- The singular behavior of the tacky-surface replica (EF10), which oscillates without any periodic contact. Vibrations occur around an equilibrium position, but the vocal folds never touch. By contrast, all other replicas with smooth surfaces vibrate with periodic collisions at the perceived pitch frequency. These observations are reminiscent of other singular vibratory behaviors already observed in excised human larynges, notably the reduction in glottal closure after vocal-fold de-epithelialization, which sometimes generated non-contact vibration of the folds^109^.
- A surface wave propagation limited to the medial-lateral direction for all silicone folds, with a reduced phase difference in the motions of the lower and upper margins. This is similar to what can be observed or simulated in the case of head/falsetto register (laryngeal mechanism M2 where only vocal-fold superficial layer vibrates)^110^.
- The cyclic pattern of the gelatin-based candidate exhibiting a “diamond-shaped” glottal aperture (see zoomed-in area in red), i.e., an index of the typical phase delay observed between the free edges of native vocal folds during modal voice^72,111^. For this hydrogel candidate, the phase difference in the motions of lower and upper margins is greater than for silicones, closer to physiology and evidenced on kymograms by the sharpness of lateral peaks^112^. This observation is noteworthy for a material whose properties have been optimized purposely to approximate the mechanics of real vocal-fold tissues under quasi-static loadings^48^.

Some of these qualitative trends were also confirmed quantitatively. In particular, Fig. 4 demonstrates that for the same flow rate, the softest and smooth-surface replica (i.e., with hydrogel) opens much more at the glottis than all the others, by a maximal ratio close to 3 at $\phi$ $\approx$ 2 L/s for instance. This result is in line with previous numerical simulations showing that increasing the vocal-fold stiffness parameters decreases their displacement and strain in the medial-lateral direction^43^, together with their closing velocity. On the contrary, the softest and tacky-surface replica (i.e., EF10) presents the smaller apertures during vibration. This demonstrates the critical role played by adhesion forces on vocal-fold vibration, which to our knowledge has never been studied in vitro nor been the subject of a dedicated numerical study, although controlling the surface condition of artificial vocal folds has already been identified as a real experimental challenge^113^.

#### Impact on the aerodynamic behavior

This is evidenced in Fig. 5a,b. Supplementary Figs. S2d,f and S3d,f also report these parameters in function of the subglottal pressure $P_s$ and the material tensile stiffness $E_t$ respectively. Several observations emerge from these figures:

- First, although the overall mechanical properties of EF10 silicone are very similar to those of the gelatin-based hydrogel in the investigated strain range (Fig. 7a–c), the differences of interface quality are such that the EF10 replica shows a very singular pressure–flow behavior compared to the one molded in hydrogel (and to those made of the two other silicones as well). More specifically, the EF10 vocal folds require a much higher flow rate to oscillate (i.e., $\phi$ > 1.5 L/s) than the hydrogel, which vibrates in the $\phi$-range from 0.5 to 2.5 L/s (Fig. 5a). Thus, whatever their level of pre-strain, the phonation threshold pressures of the EF10 vocal folds lie around 2000 Pa (i.e., in the extreme physiological range reached at high vocal intensities), whereas they all remain within the standard range between 500 Pa and 2000 Pa for hydrogels (Figs. 5a, S2f for instance). A possible scenario in view of the recorded high-speed sequences is the following: (i) at low flow rates, the initial glottal opening is still very small, close to that of all unloaded replicas (Figs. 2, 4). In such a geometric configuration, the EF10 vocal folds are sufficiently close to be sucked towards each other at the first passage of air, then remaining in contact with each other due to their sticky interface (Supplementary Videos of Fig. 2, e.g., first $\phi$-step of EF10-eps23.mp4 at the vibration onset). The subglottal pressure then rises, reaching even peaks of 8000 Pa to separate them. Not having been able to store enough elastic energy due to their rather low mechanical stiffness and stress level, they remain separated without being able to return to each other. (ii) at much higher flow rates but also much higher pre-strain ($\varepsilon _{ap}^p$ $\approx$ 0.23), the pre-stress level and stored mechanical energy are enhanced, the initial glottal area also increases (Fig. 2) so that fluid/structure interactions can yield possible sustained vibrations. However, the aerodynamic forces at the walls are so high (see pressure levels in Figs. 5a and S2f) that the elastic restoring forces are no longer sufficient to bring the folds closer to periodic contact.
- Then, leaving aside the specific trends observed for the tacky-surface material (EF10), the data show that:
  (i) the stiffer the material, the higher the subglottal pressure $P_s$ measured during the oscillations, as shown in Figs. 5a and S2f: $P_s$ values averaged for all $\phi$- and $\varepsilon _{ap}^p$-levels combined are measured around 1200 Pa, 2900 Pa and 3600 Pa for the hydrogel, EF30, and EF50 respectively. Besides, for a given material and flow rate, the higher the applied pre-strain $\varepsilon _{ap}^{p}$, the higher the pressure in the subglottal stage required to maintain self-oscillation (Figs. 5a, S2f). Typically, at $\phi \approx$ 0.7 L/s and $\varepsilon _{ap}^{p}$ $\approx$ 0.30, an increase of about 625 Pa, 800 Pa, and 1150 Pa is induced for the hydrogel, EF30, and EF50 replicas respectively, with respect to the value required for the onset of oscillations in the least deformed configuration ($\varepsilon _{ap}^{p}$ $\approx$ 0.05 ± 0.02).
  (ii) material properties of the vocal folds have also a large impact on the glottal resistance $R_g$. This is particularly evidenced at lowest airflow values, i.e., up to $\phi$ $\approx$ 2.5 L/s (Fig. 5b). Beyond this critical flow rate, $R_g$ tends towards asymptotic limits around 1500 Pa s/L, which are quite close for all the selected materials, although ranked in ascending order with material stiffness $E_t$ and pre-stress. Note that for the stiffest materials (EF30 and EF50), the resistance to the flow can reach up to 3–4 times this asymptotic value (at largest pre-strains and lowest flow rates, i.e., for $\phi \,<$ 0.5 L/s).
  These results are in line with Fig. 4 showing that for the same flow rate, the stiffer materials open much less at the glottis, resulting in increased subglottal pressure and greater resistance to airflow as measured by Alipour et al.^82^ with ex vivo measurements on several animal larynges.

#### Impact on the produced sound

Such an impact results from the complex combination of the stress–strain behavior of the material under multiple loading directions, its pre-load state, its tangent moduli (e.g., $E_t$, $G_t$), its properties of adhesion, but also of the fluid/structure coupling and possible collisions established during the vibration. In the end, the three main audio quantifiers studied in this work (*SPL*, $f_o$, and *HNR*) find themselves modified, not all in the same way, and with sometimes unexpected tendencies:

- Overall, the evolution of sound intensity according to the materials tested remains of second order to that implied by the airflow $\phi$, as shown in Fig. 5c. Increasing the initial pre-strain applied to the folds has a moderate direct effect on the *SPL*. Figure S3a also demonstrates that no direct correlation appears between the stiffness of the material and the achieved *SPL*. However, Fig. S2a clearly shows that by playing with the material properties of the replica, the same level of *SPL* can be reached over several orders of magnitude of subglottal pressure (e.g., *SPL* $\approx$ 55 dB for at least 6 distinct material and/or mechanical conditions, $P_s$ varying from $\approx$ 500 to 4000 Pa). Although less critical than flow rate, this highlights the importance of the biomechanical parameters of the system on the sound intensity, as an indirect consequence of their strong impact on the pressure distribution in the larynx.
- The variation of the acoustic fundamental frequency $f_o$ with the selected material is demonstrated in Fig. 6. Leaving aside replica EF10, whose singular interface properties induce a very complex coupling with the fluid (see above), the results show that:
  (i) focusing first on the least pre-deformed comparable configurations, the stiffer the material, the higher the $f_o$ frequency, with a discrepancy of about 20 Hz between the hydrogel and the EF50 replica for all flow rates (Fig. 6c). Based on previous sparse ex vivo^53^ and in vitro evidences^25^ as well as numerical predictions^39,43^, this increase of phonation frequency with material stiffness is expected – even though all absolute values of $f_o$ measured in the present work are very low compared to the ones encountered in standard male/female modal speech.
  (ii) pre-stretching the folds has a decreasing impact on $f_o$ for all replicas, as shown in Fig. 6d. However, this decrease remains minor, of the order of a few Hz for all materials. The maximum drop is measured around 5 Hz for the hydrogel, vibrating from 60 Hz down to 55 Hz when progressively elongated up to $\varepsilon _{ap}^p \approx$ 0.30. Such unexpected strain-induced variations are at odds with in vivo observations on real human vocal folds during glissando, where $f_o$ increases with $\varepsilon _{ap}^p$. Nor are they consistent with very recent experiments carried out on excised animal larynges, instrumented to monitor longitudinal elongation of the folds^53^, in which an increase in phonation frequency was also measured with pre-strain $\varepsilon _{ap}^p$ – albeit in a region of lower values up to 0.06 at most. However, our results are in agreement with measurements made by Shaw et al.^26^ on synthetic vocal-fold models with quasi-linear and isotropic mechanical properties, for which they showed that the $f_o$ frequency decreased slightly with fold elongation.
  These non-physiological tendencies can be explained by several discrepancies that remain between the histo-mechanical characteristics of a biological larynx and our current in vitro idealization: firstly, the longitudinal tensile response of all the materials studied in this work is still quite far from that of native vocal-fold tissues, even for the optimized hydrogel, due to its isotropy. In particular, the non-linear strain-hardening of tangent moduli observed on excised human^34,59,114^ or animal vocal folds^52,53^, which is linked to the progressive recruitment, deployment and reorientation of collagen fibres towards the load direction^34,35,49,115^, is not yet reproduced^26,48^; then, the laryngeal envelope and induced boundary conditions are probably still too soft to mimic the stiffness of native cartilages; the “active” hardening of the *vocalis* during its contraction in vivo is also left out of the current replica.
- Finally, the sound quality produced by the four candidates is affected. This is demonstrated by the harmonic-to-noise ratio (*HNR*) reported in Fig. 5d, as well as by the audio-video files associated to the spectrograms and spectra in Supplementary Figs. S4–S5. Most particularly, the sound quality produced by the EF10 replica differs greatly from that produced by the hydrogel replica. The energy levels of the sound harmonics can be up to 40 times higher than those of the background noise when the hydrogel is vibrating (Fig. 5d). Replica EF10, on the other hand, shows levels only slightly higher than the background noise. This singular noisy acoustic production of the EF10 replica (Supplementary audio file S4.d) is linked to the lack of contact between the vocal folds: incomplete glottal closure usually results in the production of a breathy voice^3,109,116^. However, whatever the selected materials, the orders of magnitude recorded for *HNR* are rather extreme compared to previous measurements in humans^102,103^. These two studies reported average values of about (i) 25 dB (resp. 20 dB) for healthy (resp. pathological) speakers, and (ii) 8 dB for young adults compared to 5 dB for elderly people. This indicates that the present materials slightly extend the range achieved in human cases with results in terms of *HNR* level below (for the silicone replicas) and above (for the hydrogel) clinical measurements (see Table 1). The hydrogel is therefore closer to non-pathological cases, unlike silicones which require higher subglottal pressure to oscillate, hence producing a higher noise level. The efficiency of hydrogel compared to silicones in producing sound with a lower noise level is remarkable, since both lower airflow and subglottal pressure are required to obtain a higher level of harmonics (above 30 dB of *HNR*) in the emitted sound.

## Conclusion

An original articulated testbed of human larynx has been developed, allowing to control the pre-phonatory posturing and degree of longitudinal pre-strain of artificial vocal folds with adjustable material properties upon finite strains. Four vocal-fold replicas were characterized, identical in geometry but molded with different silicone elastomers or with a gelatin-based hydrogel, recently optimized to approximate the mechanical behavior of native vocal folds in tension, compression and shear ex vivo^48^. This work compares the intrinsic mechanical properties of the chosen materials (stress–strain response under various loadings in finite strains, related tangent moduli, interface properties) in relation to the aerodynamic and acoustic parameters measured during the vibration of the artificial vocal folds.

The results show the ability of these expandable and isotropic vocal-fold replicas to achieve flow-induced self-oscillations over a wide range of airflow rates (from 340 mL/s to 3.5 L/s) and for several pre-strains applied in the anterior–posterior direction (up to 30$\%$). The aerodynamic and acoustic characteristics of the vibrating replicas varied according to the levels of pre-strain, in line with previous work^26,50^, and over a specific range depending on the materials. Global trends in aero-acoustic behavior were also evidenced.

The fundamental frequency of oscillation, between 50 and 80 Hz depending on the material, proved to be lower than the frequency expected for human speech, due to the geometry chosen (long and thick folds). It varied neither with airflow rate nor with subglottal pressure, in line with in vitro observations, but far from ex vivo and in vivo measurements. For the silicone vocal folds, except the tacky-surface material EF10, the fundamental frequency of oscillation increased with increasing stiffness.

A linear relationship was evidenced between airflow rate and subglottal pressure (ranging from 0.5 to 5 kPa), whose slope - namely the differentiated glottal flow resistance - remained constant for all materials (mean values ranging from 502 to 560 *Pa* *s*/*L*) except for EF10 (185 *Pa* *s*/*L*). Glottal flow resistance decreased mainly with increasing airflow rate and subglottal pressure, in relation to increased glottal width and glottal area. It increased slightly with increasing pre-strain. Subglottal pressure for hydrogel and sound pressure level for all materials are comparable with previous research based on artificial vocal folds^11^ as well as excised larynges^2,92,95,97^.

The impact of both the overall mechanical behavior and the surface adhesion properties of each tested material was evidenced and quantified with regards to the onset of the vocal-fold vibrations, the sustained vibratory pattern (in terms of glottal opening, vibratory amplitude achieved during the oscillation cycle, and contact properties between the folds), glottal flow resistance and produced sound. In particular, the performance of the hydrogel replica in comparison to the classical silicone ones is closer to human characteristics in terms of subglottal pressure to achieve phonation. In turn, the produced sound has a lower noise level, i.e., a higher harmonic-to-noise ratio, making this material an interesting candidate for further research. Moreover, the surface adhesion properties of the materials appear to play a critical role on vocal-fold vibrations. Silicone with a very tacky surface displayed very singular vibrations, requiring higher longitudinal pre-strains and subglottal pressure to self-oscillate, without any contact. This, to our knowledge, has never been studied in vitro and should be further investigated in future work.

Overall, the range of multi-physical data measured (geometrical, mechanical, and aerodynamic) matches those reported in previous in vitro studies It covers some of the ranges reported in previous in vivo and ex vivo measurements. However, it is noted that the range of certain parameters are too low (fundamental frequency $f_o<60$ Hz, part of the glottal flow resistance $R_g<3$ kPa s/L) or too high (airflow rate $\phi >0.3$ L/s, part of the maximal glottal area $A_g^{max}>100$ mm^2^) to match physiological in vivo data. Further developments are needed to improve the testbed, and design materials with greater biomimetic properties. One key limitation of the study is the nature of our current synthetic vocal folds made of a simplified, single-layered structure filled with homogeneous materials, which are still unable to reproduce the highly non-linear mechanical behavior of native vocal folds under finite-strains tension. The embedding of fibrous reinforcement with suitable microstructure gradients in the upper layers^49^ should allow to approach the J-shaped anisotropic target response in tension, and extend the phonation capabilities of artificial replicas to bring them closer to physiological ranges. Also, the use of more rigid lateral boundaries on the laryngeal envelope, such as a stiffer cartilaginous glottal stage casing, would enable more physiological geometry and vibratory patterns to be achieved.

For comparison with measured data, a simple analytical model based on Bernoulli’s principle was applied to check whether maximum glottal areas could be predicted with airflow as an input parameter. This model gave qualitatively correct trends, but it failed to accurately predict the measurements due to its strong simplifying assumptions. A next step will be the development of a more sophisticated modeling approach to gain a better understanding of the underlying physical phenomena.

A testbed that can dynamically stretch biomimetic vocal folds is very useful for exploring source-filter interaction phenomena in vitro. The next steps in the developement of the testbed will be (i) to refine the geometry of the vocal folds so as to produce pitches typical of speech and singing, (ii) to control laryngeal articulatory movements of adduction, abduction and lateral compression, (iii) to acoustically load the laryngeal tract with a vocal tract whose geometry can be adjusted according to the vowels in speech.

### Supplementary Information

Supplementary Information.

Supplementary video.

Supplementary Figure 2.

Supplementary Figure 3.

Supplementary Figure S4.

## Data Availability

The datasets generated and/or analysed during the current study ($\approx$ 4 Go + video data provided in supplementary materials) are available from the corresponding author upon request.

## References

[1] Titze IR (1988). The physics of small-amplitude oscillation of the vocal folds. J. Acoust. Soc. Am..

[2] Titze IR (2000). Principles of Voice Production.

[3] Zhang Z (2016). Mechanics of human voice production and control. J. Acoust. Soc. Am..

[4] Švec JG, Schutte HK, Chen CJ, Titze IR (2021). Integrative insights into the myoelastic-aerodynamic theory and acoustics of phonation. Scientific Tribute to Donald G. Miller. J. Voice.

[5] Herzel H (1993). Bifurcations and chaos in voice signals. Appl. Mech. Rev..

[6] Titze, I., Baken, R. J. & Herzel, H. Evidence of chaos in vocal fold vibration. In *Vocal Fold Physiology: Frontiers in Basic Science. * 143–188 (Singular Publishing Group, San Diego, CA, 1993).

[7] Lucero JC (1999). Computation of the harmonics-to-noise ratio of a voice signal using a functional data analysis algorithm. J. Sound Vib..

[8] Lucero JC (2005). Oscillation hysteresis in a two-mass model of the vocal folds. J. Sound Vib..

[9] Jiang JJ, Zhang Y, McGilligan C (2006). Chaos in voice, from modeling to measurement. J. Voice.

[10] Van den Berg J, Zantema J, Doornenbal P (1957). On the air resistance and the Bernoulli effect of the human larynx. J. Acoust. Soc. Am..

[11] Kniesburges S (2011). In vitro experimental investigation of voice production. Curr. Bioinform..

[12] Scherer RC (2001). Intraglottal pressure profiles for a symmetric and oblique glottis with a divergence angle of 10 degrees. J. Acoust. Soc. Am..

[13] Barney AM, Shadle C, Davies P (1999). Fluid flow in a dynamic mechanical model of the vocal folds and tract. I. Measurements and theory. J. Acoust. Soc. Am..

[14] Deverge M (2003). Influence of collision on the flow through in-vitro rigid models of the vocal folds. J. Acoust. Soc. Am..

[15] Cisonni J, Van Hirtum A, Pelorson X, Willems J (2008). Theoretical simulation and experimental validation of inverse quasi-one-dimensional steady and unsteady glottal flow models. J. Acoust. Soc. Am..

[16] Thomson SL, Mongeau L, Frankel SH (2005). Aerodynamic transfer of energy to the vocal folds. J. Acoust. Soc. Am..

[17] Zhang Z, Neubauer J, Berry DA (2006). Aerodynamically and acoustically driven modes of vibration in a physical model of the vocal folds. J. Acoust. Soc. Am..

[18] Zhang Z, Neubauer J, Berry D (2006). The influence of subglottal acoustics on laboratory models of phonation. J. Acoust. Soc. Am..

[19] Ruty N, Pelorson X, Hirtum AV, Lopez-Arteaga I, Hirschberg A (2007). An in vitro setup to test the relevance and the accuracy of low-order vocal folds models. J. Acoust. Soc. Am..

[20] Drechsel JS, Thomson SL (2008). Influence of supraglottal structures on the glottal jet exiting a two-layer synthetic, self-oscillating vocal fold model. J. Acoust. Soc. Am..

[21] Murray PR, Thomson SL (2011). Synthetic, multi-layer, self-oscillating vocal fold model fabrication. J. Vis. Exp..

[22] Murray PR, Thomson SL (2012). Vibratory responses of synthetic, self-oscillating vocal fold models. J. Acoust. Soc. Am..

[23] Titze IR, Schmidt SS, Titze MR (1995). Phonation threshold pressure in a physical model of the vocal fold mucosa. J. Acoust. Soc. Am..

[24] Pickup BA, Thomson SL (2009). Influence of asymmetric stiffness on the structural and aerodynamic response of synthetic vocal fold models. J. Biomech..

[25] Mendelsohn AH, Zhang Z (2011). Phonation threshold pressure and onset frequency in a two-layer physical model of the vocal folds. J. Acoust. Soc. Am..

[26] Shaw SM, Thomson SL, Dromey C, Smith S (2012). Frequency response of synthetic vocal fold models with linear and nonlinear material properties. J. Speech Lang. Hear. Res..

[27] Xuan Y, Zhang Z (2014). Influence of embedded fibers and an epithelium layer on the glottal closure pattern in a physical vocal fold model. J. Speech Lang. Hear. Res..

[28] Luizard P, Pelorson X (2017). Threshold of oscillation of a vocal fold replica with unilateral surface growths. J. Acoust. Soc. Am..

[29] Motie-Shirazi M (2023). Effect of nodule size and stiffness on phonation threshold and collision pressures in a synthetic hemilaryngeal vocal fold model. J. Acoust. Soc. Am..

[30] Alipour F (2011). Mathematical models and numerical schemes for the simulation of human phonation. Curr. Bioinform..

[31] Döllinger M (2023). Overview on state-of-the-art numerical modeling of the phonation process. Acta Acust..

[32] Titze IR, Hunter EJ (2004). Normal vibration frequencies of the vocal ligament. J. Acoust. Soc. Am..

[33] Zhang K, Siegmund T, Chan RW (2007). A two-layer composite model of the vocal fold lamina propria for fundamental frequency regulation. J. Acoust. Soc. Am..

[34] Kelleher J, Siegmund T, Du M, Naseri E, Chan R (2013). The anisotropic hyperelastic biomechanical response of the vocal ligament and implications for frequency regulation: A case study. J. Acoust. Soc. Am..

[35] Terzolo A, Bailly L, Orgéas L, Cochereau T, Bernardoni NH (2022). A micro-mechanical model for the fibrous tissues of vocal folds. J. Mech. Behav. Biomed. Mater..

[36] Zhang Z (2009). Characteristics of phonation onset in a two-layer vocal fold model. J. Acoust. Soc. Am..

[37] Zhang Z (2010). Dependence of phonation threshold pressure and frequency on vocal fold geometry and biomechanics. J. Acoust. Soc. Am..

[38] Zhang Z (2014). The influence of material anisotropy on vibration at onset in a three-dimensional vocal fold model. J. Acoust. Soc. Am..

[39] Zhang Z (2016). Cause-effect relationship between vocal fold physiology and voice production in a three-dimensional phonation model. J. Acoust. Soc. Am..

[40] Zhang Z (2017). Effect of vocal fold stiffness on voice production in a three-dimensional body-cover phonation model. J. Acoust. Soc. Am..

[41] Xue Q, Zheng X, Mittal R, Bielamowicz S (2014). Computational study of effects of tension imbalance on phonation in a three-dimensional tubular larynx model. J. Voice.

[42] Pham N, Xue Q, Zheng X (2018). Coupling between a fiber-reinforced model and a Hill-based contractile model for passive and active tissue properties of laryngeal muscles: A finite element study. J. Acoust. Soc. Am..

[43] Wang X, Jiang W, Zheng X, Xue Q (2021). A computational study of the effects of vocal fold stiffness parameters on voice production. J. Voice.

[44] Wang X, Zheng X, Xue Q (2023). The influence of fiber orientation of the conus elasticus in vocal fold modeling. J. Biomech. Eng..

[45] Greenwood TE, Thomson SL (2021). Embedded 3D printing of multi-layer, self-oscillating vocal fold models. J. Biomech..

[46] Häsner P, Birkholz P (2023). Reproducibility and aging of different silicone vocal folds models. J. Voice.

[47] Schmidt B (2013). Material and shape optimization for multi-layered vocal fold models using transient loadings. J. Acoust. Soc. Am..

[48] Yousefi-Mashouf H, Bailly L, Orgéas L, Henrich Bernardoni N (2023). Mechanics of gelatin-based hydrogels during finite strain tension, compression and shear. Front. Bioeng. Biotechnol..

[49] Ferri-Angulo D (2023). Versatile fiber-reinforced hydrogels to mimic human vocal-fold microstructure and mechanics. Acta Biomater..

[50] Tur B (2023). Effect of ligament fibers on dynamics of synthetic, self-oscillating vocal folds in a biomimetic larynx model. Bioengineering.

[51] Shariati A, Wurdemann HA (2023). Analysis of a soft bio-inspired active actuation model for the design of artificial vocal folds. J. Mech. Robot..

[52] Lamprecht R (2022). Quasi-static ultrasound elastography of ex-vivo porcine vocal folds during passive elongation and adduction. J. Voice.

[53] Scheible F (2023). Behind the complex interplay of phonation: Investigating elasticity of vocal folds with pipette aspiration technique during ex vivo phonation experiments. J. Voice.

[54] Murray PR, Thomson SL, Smith ME (2014). A synthetic, self-oscillating vocal fold model platform for studying augmentation injection. J. Voice.

[55] Goodyer E (2011). Devices and methods on analysis of biomechanical properties of laryngeal tissue and substitute materials. Curr. Bioinform..

[56] Miri AK (2014). Mechanical characterization of vocal fold tissue: A review study. J. Voice.

[57] Lagier A (2017). Control of the glottal configuration in ex vivo human models: Quantitative anatomy for clinical and experimental practices. Surg. Radiol. Anat..

[58] Bailly L (2018). 3D multiscale imaging of human vocal folds using synchrotron X-ray microtomography in phase retrieval mode. Sci. Rep..

[59] Cochereau T (2020). Mechanics of human vocal folds layers during finite strains in tension, compression and shear. J. Biomech..

[60] Bailly L, Pelorson X, Henrich N, Ruty N (2008). Influence of a constriction in the near field of the vocal folds: Physical modeling and experimental validation. J. Acoust. Soc. Am..

[61] Latifi N (2016). A flow perfusion bioreactor system for vocal fold tissue engineering applications. Tissue Eng. Part C Methods.

[62] Weiss S, Sutor A, Ilg J, Rupitsch SJ, Lerch R (2016). Measurement and analysis of the material properties and oscillation characteristics of synthetic vocal folds. Acta Acust. United Acust..

[63] Fuller KNG, Tabor D (1975). The effect of surface roughness on the adhesion of elastic solids. Proc. R. Soc. Lond. Ser. A Math. Phys. Sci..

[64] Lorenz B (2013). Adhesion: Role of bulk viscoelasticity and surface roughness. J. Phys. Condens. Matter.

[65] De Cheveigné A, Kawahara H (2002). YIN, a fundamental frequency estimator for speech and music. J. Acoust. Soc. Am..

[66] Agarwal, M. *The false vocal folds and their effect on translaryngeal airflow resistance*. Ph.D. Thesis, Bowling Green State University (2004).

[67] Xue Q, Zheng X (2017). The effect of false vocal folds on laryngeal flow resistance in a tubular three-dimensional computational laryngeal model. J. Voice.

[68] Birk V (2017). Influence of glottal closure on the phonatory process in ex vivo porcine larynges. J. Acoust. Soc. Am..

[69] Sadeghi H, Döllinger M, Kaltenbacher M, Kniesburges S (2019). Aerodynamic impact of the ventricular folds in computational larynx models. J. Acoust. Soc. Am..

[70] Kist AM (2021). A deep learning enhanced novel software tool for laryngeal dynamics analysis. J. Speech Lang. Hear. Res..

[71] Van Hirtum A, Bouvet A, Tokuda I, Pelorson X (2022). Dynamic vibration mode decomposition of auto-oscillating vocal fold replicas without and with vertical tilting. J. Sound Vib..

[72] Švec JG, Schutte HK (1996). Videokymography: High-speed line scanning of vocal fold vibration. J. Voice.

[73] Van den Berg J, Zantema J, Doornenbal P (1957). On the air resistance and the Bernoulli effect of the human larynx. J. Acoust. Soc. Am..

[74] Ishizaka K, Flanagan JL (1972). Synthesis of voiced sounds from a two-mass model of the vocal cords. Bell Syst. Tech. J..

[75] Pelorson X, Hirschberg A, van Hassel RR, Wijnands APJ, Auregan Y (1994). Theoretical and experimental study of quasi steady-flow separation within the glottis during phonation. Application to a modified two-mass model. J. Acoust. Soc. Am..

[76] Pelorson X (2001). On the meaning and accuracy of the pressure–flow technique to determine constriction areas within the vocal tract. Speech Commun..

[77] Decker GZ, Thomson SL (2007). Computational simulations of vocal fold vibration: Bernoulli versus Navier–Stokes. J. Voice.

[78] Patel RR, Sundberg J, Gill B, Lã FM (2022). Glottal airflow and glottal area waveform characteristics of flow phonation in untrained vocally healthy adults. J. Voice.

[79] Horáček J, Laukkanen A-M, Šidlof P, Murphy P, Švec JG (2009). Comparison of acceleration and impact stress as possible loading factors in phonation: A computer modeling study. Folia Phoniatr. Logop..

[80] Smith ME, Green DC, Berke GS (1991). Pressure–flow relationships during phonation in the canine larynx. J. Voice.

[81] Alipour F, Scherer RC, Finnegan E (1997). Pressure–flow relationships during phonation as a function of adduction. J. Voice.

[82] Alipour F, Jaiswal S (2009). Glottal airflow resistance in excised pig, sheep, and cow larynges. J. Voice.

[83] Birk V, Sutor A, Döllinger M, Bohr C, Kniesburges S (2016). Acoustic impact of ventricular folds on phonation studied in ex vivo human larynx models. Acta Acust. United Acust..

[84] Döllinger M, Berry DA, Kniesburges S (2016). Dynamic vocal fold parameters with changing adduction in ex-vivo hemilarynx experiments. J. Acoust. Soc. Am..

[85] Titze IR, Martin DW (1998). Principles of Voice Production.

[86] Coleman RF, Mabis JH, Hinson JK (1977). Fundamental frequency-sound pressure level profiles of adult male and female voices. J. Speech Hear. Res..

[87] Tanaka S, Gould WJ (1983). Relationships between vocal intensity and noninvasively obtained aerodynamic parameters in normal subjects. J. Acoust. Soc. Am..

[88] Björklund S, Sundberg J (2016). Relationship between subglottal pressure and sound pressure level in untrained voices. J. Voice.

[89] Döllinger M, Berry DA, Luegmair G, Hüttner B, Bohr C (2012). Effects of the epilarynx area on vocal fold dynamics and the primary voice signal. J. Voice.

[90] Motie-Shirazi M (2022). Collision pressure and dissipated power dose in a self-oscillating silicone vocal fold model with a posterior glottal opening. J. Speech Lang. Hear. Res..

[91] Schutte HK (1980). The Efficiency of Voice Production.

[92] Švec JG, Granqvist S (2018). Tutorial and guidelines on measurement of sound pressure level in voice and speech. J. Speech Lang. Hear. Res..

[93] Henrich N (2006). Mirroring the voice from Garcia to the present day: Some insights into singing voice registers. Logop. Phoniatr. Vocol..

[94] Roubeau B, Henrich N, Castellengo M (2009). Laryngeal vibratory mechanisms: The notion of vocal register revisited. J. Voice.

[95] Baken R, Orlikoff RF (2000). Clinical Measurement of Speech and Voice.

[96] Murray PR, Thomson SL (2012). Vibratory responses of synthetic, self-oscillating vocal fold models. J. Acoust. Soc. Am..

[97] Lehoux H, Hampala V, Švec JG (2021). Subglottal pressure oscillations in anechoic and resonant conditions and their influence on excised larynx phonations. Sci. Rep..

[98] Carroll LM (1996). Respiratory and glottal efficiency measures in normal classically trained singers. J. Voice.

[99] Master S, Guzman M, Azócar MJ, Muñoz D, Bortnem C (2015). How do laryngeal and respiratory functions contribute to differentiate actors/actresses and untrained voices?. J. Voice.

[100] Qi Y, Hillman RE (1997). Temporal and spectral estimations of harmonics-to-noise ratio in human voice signals. J. Acoust. Soci. Am..

[101] Yumoto E, Gould WJ, Baer T (1982). Harmonics-to-noise ratio as an index of the degree of hoarseness. J. Acoust. Soc. Am..

[102] Ferrand CT (2002). Harmonics-to-noise ratio: An index of vocal aging. J. Voice.

[103] Fernandes J, Teixeira F, Guedes V, Junior A, Teixeira JP (2018). Harmonic to noise ratio measurement-selection of window and length. Procedia Comput. Sci..

[104] Alipour F, Vigmostad S (2012). Measurement of vocal folds elastic properties for continuum modeling. J. Voice.

[105] Min YB, Titze IR, Alipour-Haghighi F (1995). Stress–strain response of the human vocal ligament. Ann. Otol. Rhinol. Laryngol..

[106] Chan R, Fu M, Young L, Tirunagari N (2007). Relative contributions of collagen and elastin to elasticity of the vocal fold under tension. Ann. Biomed. Eng..

[107] Kelleher JE, Siegmund T, Du M, Naseri E, Chan RW (2013). Empirical measurements of biomechanical anisotropy of the human vocal fold lamina propria. Biomech. Model. Mechanobiol..

[108] Henrich N, d’Alessandro C, Doval B, Castellengo M (2005). Glottal open quotient in singing: Measurements and correlation with laryngeal mechanisms, vocal intensity, and fundamental frequency. J. Acoust. Soc. Am..

[109] Tse JR, Zhang Z, Long JL (2015). Effects of vocal fold epithelium removal on vibration in an excised human larynx model. J. Acoust. Soc. Am..

[110] Kumar SP, Švec JG (2019). Kinematic model for simulating mucosal wave phenomena on vocal folds. Biomed. Signal Process. Control.

[111] Švec J, Shutte HK (2012). Kymographic imaging of laryngeal vibrations. Curr. Opin. Otolaryngol. Head Neck Surg..

[112] Kumar SP (2020). Visual and automatic evaluation of vocal fold mucosal waves through sharpness of lateral peaks in high-speed videokymographic images. J. Voice.

[113] Gabriel, F., Häsner, P., Dohmen, E., Borin, D. & Birkholz, P. Surface stickiness and waviness of two-layer silicone structures for synthetic vocal folds. In *Konferenz Elektronische Sprachsignalverarbeitung.* 221–230 (TUDpress, Dresden, 2019).

[114] Kelleher JE, Siegmund T, Chan RW, Henslee EA (2011). Optical measurements of vocal fold tensile properties: Implications for phonatory mechanics. J. Biomech..

[115] Miri AK, Heris HK, Tripathy U, Wiseman PW, Mongeau L (2013). Microstructural characterization of vocal folds toward a strain-energy model of collagen remodeling. Acta Biomater..

[116] Södersten M, Lindestad P (1990). Glottal closure and perceived breathiness during phonation in normally speaking subjects. J. Speech Hear. Res..

