# Author Correction: Flow-induced oscillations of vocal-fold replicas with tuned extensibility and material properties

**DOI:** 10.1038/s41598-024-52690-4

**Published:** 2024-01-30

**Authors:** Paul Luizard, Lucie Bailly, Hamid Yousefi-Mashouf, Raphaël Girault, Laurent Orgéas, Nathalie Henrich Bernardoni

**Affiliations:** 1 Univ. Grenoble Alpes, CNRS, Grenoble INP, GIPSA-lab, 38000 Grenoble, France; 2 Univ. Grenoble Alpes, CNRS, Grenoble INP, 3SR, 38000 Grenoble, France; 3 Present Address: CNRS, Centrale Marseille, Aix Marseille Univ, LMA UMR 7031, Marseille, France; 4 Present Address: Audio Communication Group, Technische Universität Berlin, Einsteinufer 17c, 10587 Berlin, Germany

Correction to: *Scientific Reports* 10.1038/s41598-023-48080-x, published online 19 December 2023

The original version of this Article contained errors in the Affiliations.

Affiliation 1 was incorrectly given as ‘CNRS, Grenoble INP, GIPSA-lab, Univ. Grenoble Alpes, Grenoble 38000, France’. The correct affiliation is listed below.

Univ. Grenoble Alpes, CNRS, Grenoble INP, GIPSA-lab, Grenoble, 38000, France

Additionally, Affiliation 2 was incorrectly given as ‘CNRS, Grenoble INP, 3SR, Univ. Grenoble Alpes, Grenoble 38,000, France’. The correct affiliation is listed below.

Univ. Grenoble Alpes, CNRS, Grenoble INP, 3SR, Grenoble, 38000, France

The original Article has been corrected.

