# Supplementary material for: Flow-induced oscillations of vocal-fold replicas with tuned extensibility and material properties
Source: Sci Rep. 2023 Dec 19;13:22658. doi: 10.1038/s41598-023-48080-x (PMC10730560; doi:10.1038/s41598-023-48080-x)
Supplement: Supplementary file 1 — Supplementary Information. [file 41598_2023_48080_MOESM1_ESM.pdf]

Flow-induced oscillations of vocal-fold replicas with tuned extensibility and material properties

Paul Luizard[1,+], **Lucie Bailly**[2], **Hamid Yousefi-Mashouf**[1,2], **Raphaël Girault**[1], **Laurent Orgéas**[2], **and Nathalie Henrich Bernardoni**[1,*]

[1]Univ. Grenoble Alpes, CNRS, Grenoble INP, GIPSA-lab, Grenoble, 38000, France
[2]Univ. Grenoble Alpes, CNRS, Grenoble INP, 3SR, Grenoble, 38000, France
[*]Corresponding author: nathalie.henrich@gipsa-lab.fr
[+]Current affiliations: Aix Marseille Univ, CNRS, Centrale Marseille, LMA UMR 7031, Marseille, France
Audio Communication Group, Technische Universität Berlin, Einsteinufer 17c, Berlin, D-10587, Germany

SUPPLEMENTARY INFORMATION

In addition to the Figures and Tables presented in this document, video clips and audio files are provided as Supplementary Material. Here is a description of each additional file :

- **4mat_airflow2Ls.mp4** is a video of the replicas during phonation, captured with the high speed camera. The four materials under study are presented as an example of their vibrating behavior, for specific values of airflow and strain.
- **Fig2_videos.zip** is a collection of videos of the replicas during phonation, captured with the high speed camera. They illustrate Fig. 2 of the article. For each material, the values of strain presented in Fig. 2 appear. For each of these strain values, all corresponding airflow values are covered.
- **Fig3_videos.zip** is a collection of videos of the replicas during phonation, captured with the high speed camera. They illustrate Fig. 3 of the article. For each material, the value of strain presented in Fig. 3 appears and all corresponding airflow values are covered.
- **FigS4_audios.zip** is a collection of the six audio clips corresponding to Fig S4 of the Supplementary Material document where the spectrograms are presented.

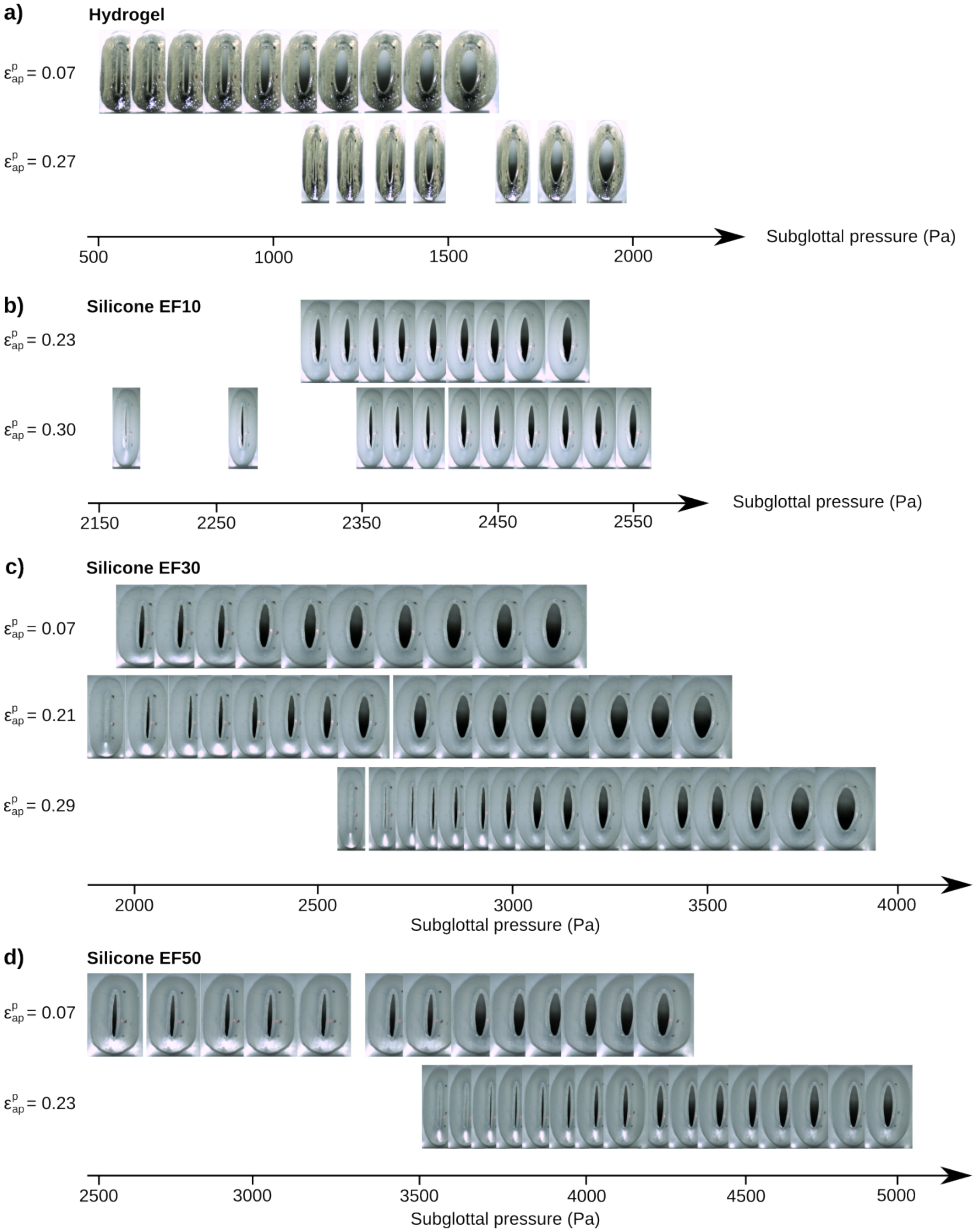

Figure S1. Pictures of the maximal glottal opening A_g^{max} achieved during oscillation for various subglottal pressure and pre-strain values with **a)** hydrogel, **b)** EF10, **c)** EF30, and **d)** EF50 silicone models. This illustrates specific series showed in Fig. 2 of the article. The blank spaces between pictures correspond to subglottal pressure values at which no measurement was performed. Corresponding videos for all pre-strains and increasing airflow sequences are provided as supplementary material.

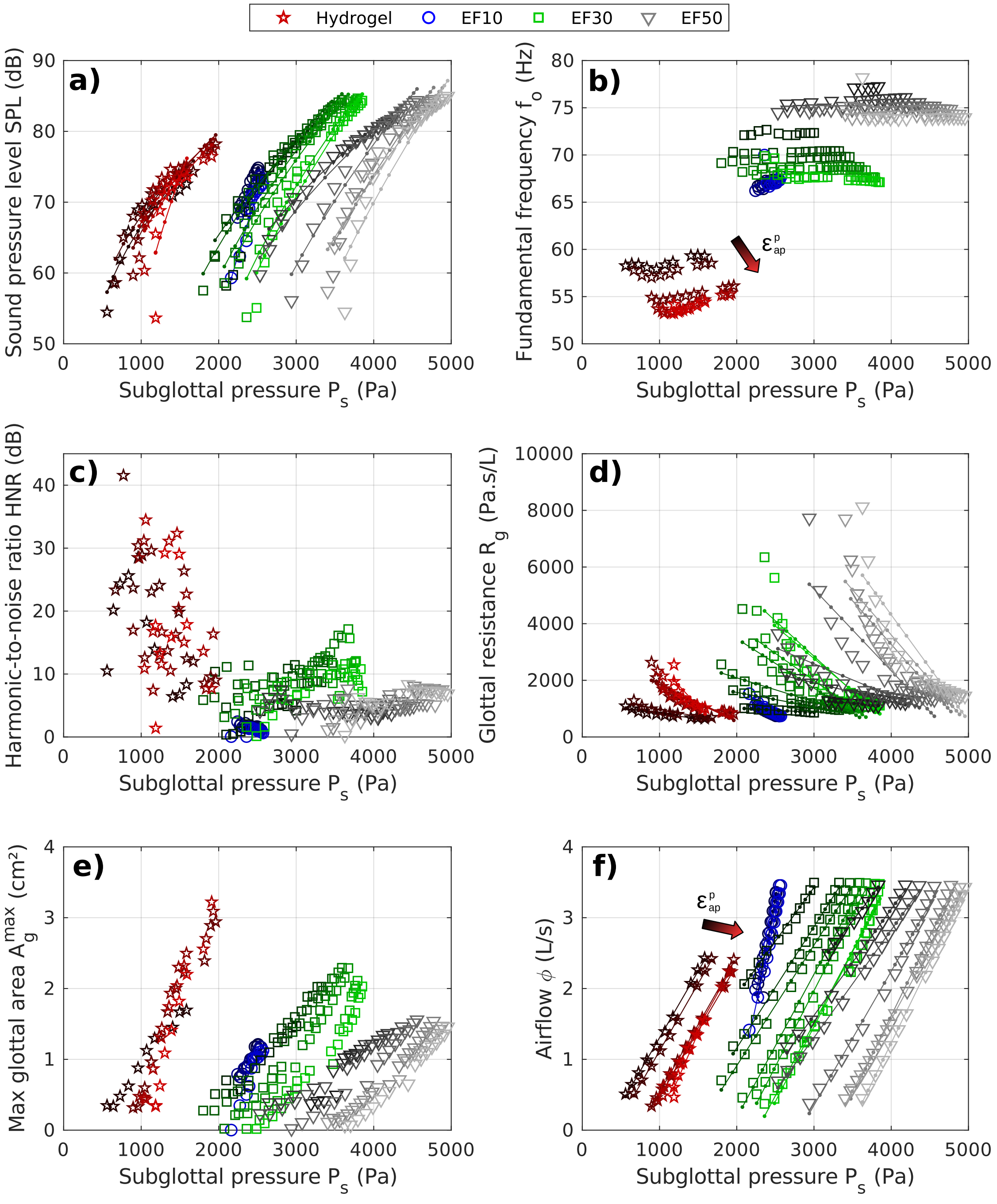

Figure S2. Audio (sound pressure level, fundamental frequency, harmonic-to-noise ratio), aerodynamic (mean glottal flow resistance, airflow), and geometric (maximal glottal area) parameters as a function of mean subglottal pressure, for each material and all pre-strain level ε_{ap}^{p} along the antero-posterior direction. Dark to light colors stand for increasing ε_{ap}^{p} values from Table S1. Markers stand for experimental data points while solid lines represent the best empirical fits adjusted to the data for a given ε_{ap}^{p}.

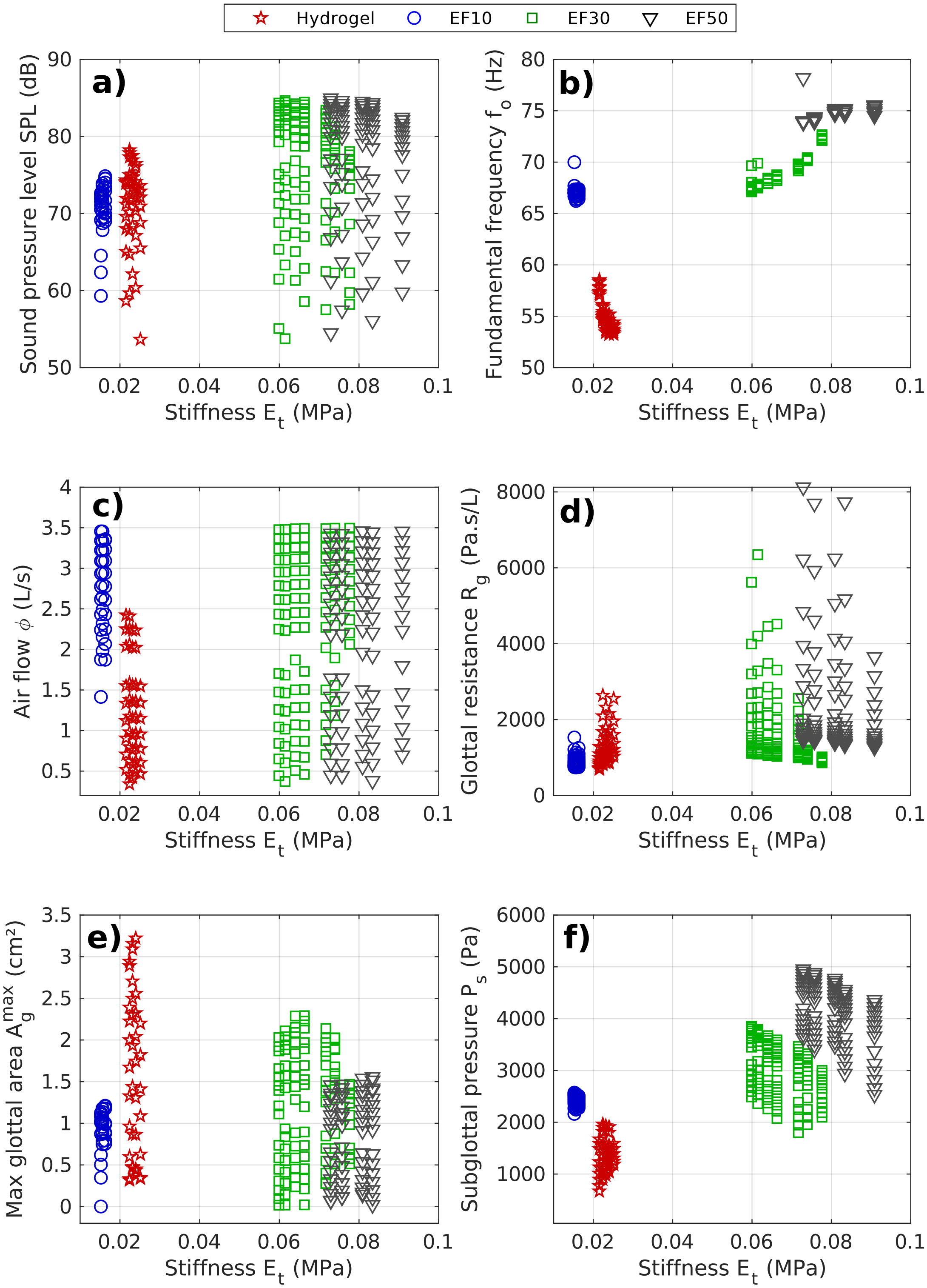

Figure S3. Geometric and aero-acoustic quantifiers of the vocal-fold vibrations against the tensile stiffness E_t of each material, at all recorded airflow and pre-strain values.

	EF 10			EF30			EF50			Hydrogel		
**Fitting model**	ε_{ap}^{p}	R_d	P_0	ε_{ap}^{p}	R_d	P_0	ε_{ap}^{p}	R_d	P_0	ε_{ap}^{p}	R_d	P_0
							0.03	694.3	1448.4	0.07	530.7	307.3
$P_s = R_d\ \phi + P_0$				0.07	652.7	759.1	0.04	663.8	1926.9	0.10	525.4	412.6
				0.10	581.1	1329.5	0.07	656.1	2144.4	0.16	522.8	721.1
with P_s in Pa,	0.23	151.6	1993.4	0.13	584.0	1473.1	0.13	523.5	2824.3	0.20	535.7	749.3
R_d in Pa.s.L^{-1},	0.27	208.5	1842.0	0.19	491.9	1916.9	0.16	444.0	3283.8	0.24	511.2	763.4
and P_0 in Pa	0.30	197.1	1901.8	0.21	465.2	2084.1	0.20	496.3	3224.5	0.27	389.4	961.0
				0.26	473.4	2292.6	0.23	445.7	3451.4			
				0.29	467.8	2318.8						
**Fitting model**	ε_{ap}^{p}	k_1	ℓ_1	ε_{ap}^{p}	k_1	ℓ_1	ε_{ap}^{p}	k_1	ℓ_1	ε_{ap}^{p}	k_1	ℓ_1
										0.07	25.1	64.1
$SPL = k_1\ \log_{10}(\phi) + \ell_1$				0.07	57.8	47.4	0.04	33.9	63.7	0.10	21.4	67.4
				0.10	36.0	62.1	0.07	31.8	66.0	0.16	20.3	71.2
with SPL in dB,	0.23	24.9	61.6	0.13	35.4	64.6	0.13	30.0	69.0	0.20	18.6	72.2
k_1 in dB.s.L^{-1},	0.27	25.5	60.2	0.19	29.5	68.4	0.16	29.8	69.4	0.24	20.9	70.5
and ℓ_1 in dB	0.30	38.1	53.1	0.21	27.7	69.3	0.20	28.4	70.0	0.27	33.6	69.7
				0.26	30.5	69.1	0.23	31.7	69.0			
				0.29	32.3	67.6						
**Fitting model**	ε_{ap}^{p}	k_2	ℓ_2	ε_{ap}^{p}	k_2	ℓ_2	ε_{ap}^{p}	k_2	ℓ_2	ε_{ap}^{p}	k_2	ℓ_2
										0.07	37	-44
$SPL = k_2\ \log_{10}(P_s) + \ell_2$				0.07	87	-224	0.04	80	-207	0.10	35	-36
				0.10	74	-179	0.07	91	-247	0.16	47	-75
with k_2 in dB.Pa^{-1}	0.23	140	-400	0.13	85	-219	0.13	137	-416	0.20	41	-57
and l_2 in dB	0.27	109	-299	0.19	102	-278	0.16	161	-506	0.24	46	-73
	0.30	194	-587	0.21	105	-289	0.20	146	-451	0.27	105	-259
				0.26	125	-363	0.23	185	-596			
				0.29	133	-391						

Table S1. Slope and y-intercept of the linear relationships between subglottal pressure P_s and airflow rate ϕ, sound pressure level SPL and ϕ or P_s, fitted to experimental values for each material and pre-strain condition ε_{ap}^{p}.

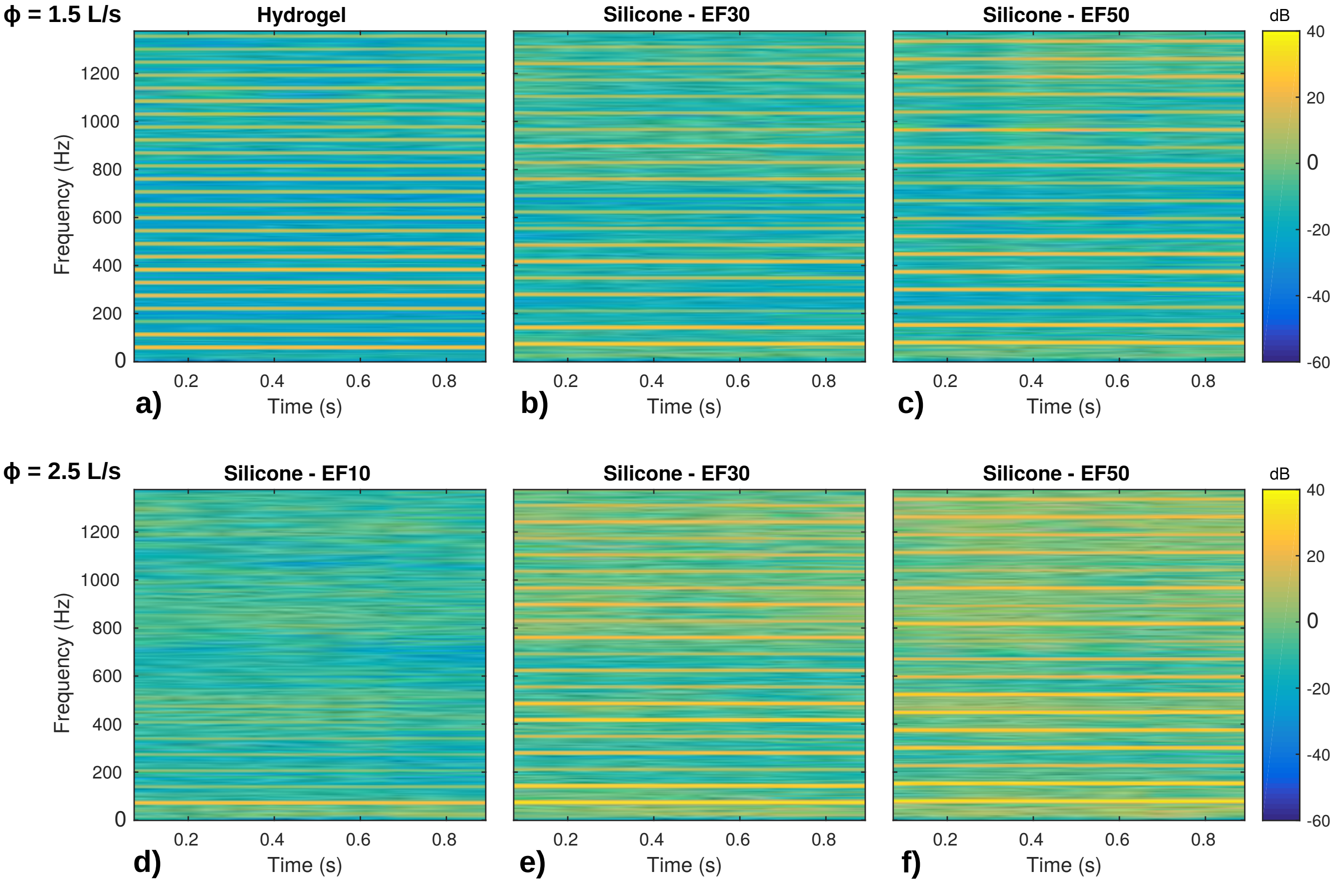

Figure S4. Spectrograms of the sound produced by different materials at a given strain value $\varepsilon_{ap}^{p} \approx 0.23$, for two different air flow values $\phi = 1.5$ and $\phi = 2.5$ L/s. The audio files are also available to listen to these sounds.

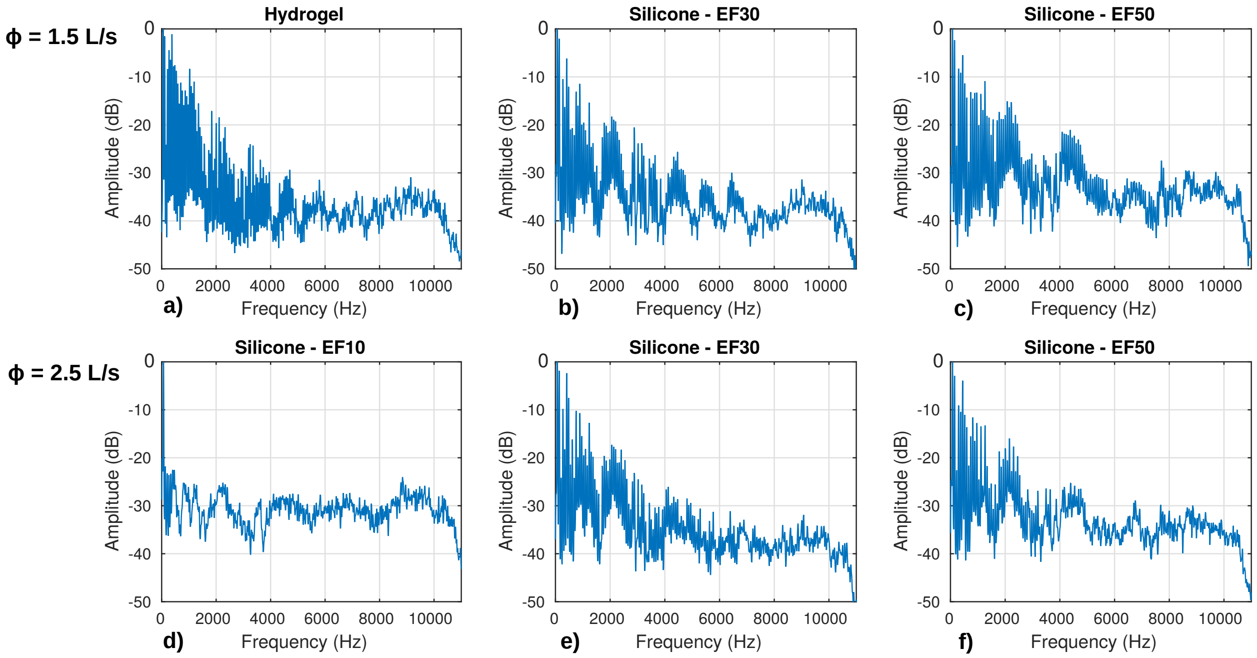

Figure S5. Long term average spectra (LTAS) of the sound produced by different materials at a given strain value $\varepsilon_{ap}^{p} \approx 0.23$, for two different air flow values $\phi = 1.5$ L/s and $\phi = 2.5$ L/s.
